# Method parameters’ impact on mortality and variability in mouse stroke experiments: a meta-analysis

**DOI:** 10.1038/srep21086

**Published:** 2016-02-15

**Authors:** Edvin Ingberg, Hua Dock, Elvar Theodorsson, Annette Theodorsson, Jakob O. Ström

**Affiliations:** 1 Division of Microbiology and Molecular Medicine, Department of Clinical and Experimental Medicine, Linköping University, Department of Clinical Chemistry, Center for Diagnostics, Region Östergötland, Sweden; 2 Division of Neuro and Inflammation Science, Department of Clinical and Experimental Medicine, Linköping University, Department of Neurosurgery, Anaesthetics, Operations and Specialty Surgery Center, Region Östergötland, Sweden; 3 Vårdvetenskapligt Forskningscentrum/Centre for Health Sciences, Örebro University Hospital, County Council of Örebro, Örebro, Sweden; 4 School of Health and Medical Sciences, Örebro University, Örebro, Sweden

## Abstract

Although hundreds of promising substances have been tested in clinical trials, thrombolysis currently remains the only specific pharmacological treatment for ischemic stroke. Poor quality, e.g. low statistical power, in the preclinical studies has been suggested to play an important role in these failures. Therefore, it would be attractive to use animal models optimized to minimize unnecessary mortality and outcome variability, or at least to be able to power studies more exactly by predicting variability and mortality given a certain experimental setup. The possible combinations of methodological parameters are innumerous, and an experimental comparison of them all is therefore not feasible. As an alternative approach, we extracted data from 334 experimental mouse stroke articles and, using a hypothesis-driven meta-analysis, investigated the method parameters’ impact on infarct size variability and mortality. The use of Swiss and C57BL6 mice as well as permanent occlusion of the middle cerebral artery rendered the lowest variability of the infarct size while the emboli methods increased variability. The use of Swiss mice increased mortality. Our study offers guidance for researchers striving to optimize mouse stroke models.

Stroke is amongst the most common causes of death and disability worldwide^1^. Major advances have been made in the understanding of the pathophysiology of stroke and *in vitro* and animal experiments have suggested numerous substances as promising candidates for treatment of the disease^2^,^3^. However, although hundreds of these substances have been tested in clinical trials, thrombolysis is still the only specific pharmacological treatment proven efficacious in acute ischemic stroke^2^. The apparent difficulty of transferring results from experimental studies to the clinical situation (“from bench to bedside”) has been referred to as a “translational roadblock”^2^,^4^ and the possible reasons behind it, particularly lack of methodological quality, has been discussed intensively over the last years^5^,^6^. Low statistical power as a results of high outcome variability and mortality in combination with a small group sizes has been suggested to be an important issue^5^ and although this can theoretically be overcome by increasing the group sizes enough, such a solution has several problematic implications. From an ethical point of view, it is recommended to use as few animals as possible according to the “three R principle”^7^ and working with large number of animals is both practically inconvenient (time and space consuming) and costly. Therefore, as a complement, it would be attractive to optimize the animal model by minimizing unnecessary outcome variability and mortality, or at least be able to power studies more exactly by predicting variability and mortality given a certain experimental setup.

Simplified, the standard approach in the majority of the preclinical stroke studies consists of three steps: 1) focal cerebral ischemia is induced in rodents, 2) some kind of treatment is administered and 3) outcome, most often by measuring infarct sizes, is assessed. These basic steps are employed in hundreds of publications each year but unfortunately no consensus exits regarding the ideal setup, and since the variations in methodological factors are innumerous, it is very complicated to experimentally evaluate all possible combinations. In an attempt to address this question, we performed a hypothesis-driven meta-analysis in 2013 studying method parameters’ impact on mortality and variability in rat stroke experiments^8^. However, since the previous study only used data from rat studies, and since mice are becoming increasingly popular in the preclinical stroke field, we decided to perform a similar analysis on mice. Thus, the objective of the current study was to investigate the effect of methodological variables on infarct size variability and mortality in mouse stroke experiments. Specifically, eight a priori hypotheses concerning factor-outcome relations were formulated:

1. Middle cerebral artery occlusion duration affects (A) infarct size variability and (B) mortality.
2. Type of focal cerebral ischemia procedure affects (A) infarct size variability and (B) mortality.
3. Mouse strain affects (A) infarct size variability and (B) mortality.
4. In studies using the intraluminal filament method, the type of occluding filament affects (A) infarct size variability and (B) mortality.

## Results

### Regression models

**Figure 1:**
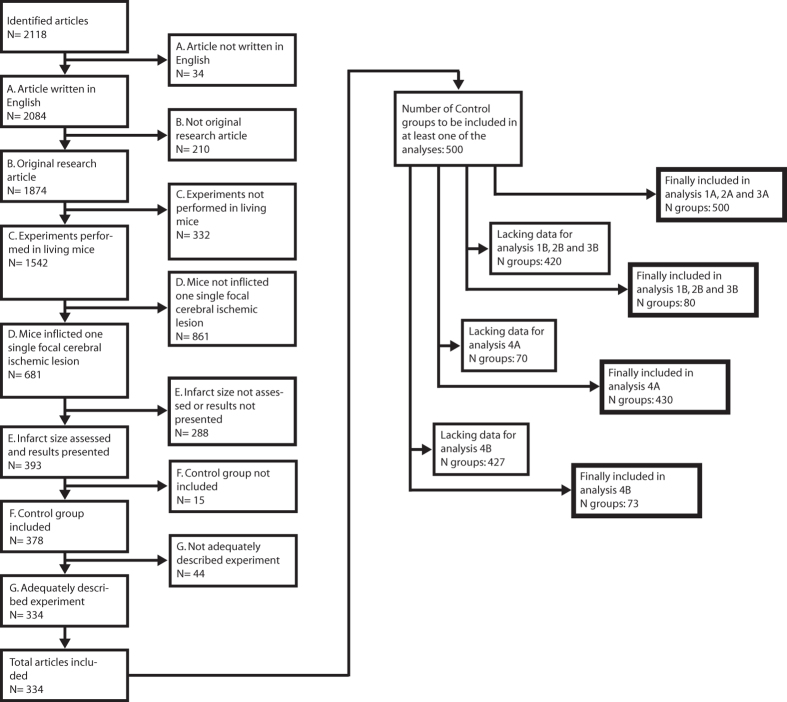
Article inclusion. A total of 2118 articles were assessed for inclusion. After exclusion according to criteria (**A–G**), 334 articles describing 500 control groups remained. All control groups could not be used for all hypotheses due to lack of essential information; the number of control groups included in each analysis are specified in the thick-boarded boxes.

The regression model addressing hypotheses 1A, 2A and 3A included 500 control groups while the analysis for hypothesis 4A included 430 (Fig. 1). The r^2^ values were 0.22 and 0.26, meaning that 22% and 26% of the variation in the outcome measures *Infarct size coefficient of variation* were explained by the models, respectively. The two models analyzing impact on *Mortality rate*, one for hypotheses 1B, 2B and 3B and one for hypothesis 4B, included 80 and 73 control groups, respectively. The resulting r^2^ values were 0.72 and 0.78.

### Impact of occlusion duration on infarct size variability and mortality (hypotheses 1A and 1B)

**Figure 2:**
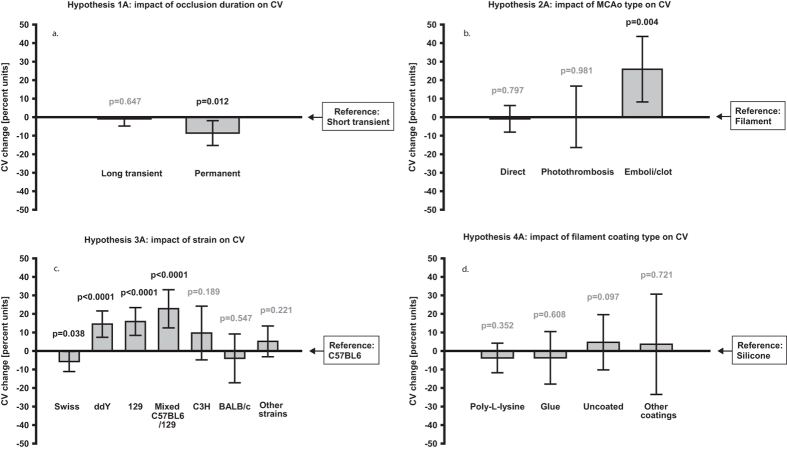
Method parameters’ impact on infarct size variability. Bars represent change in *Infarct size coefficient of variation,* measured in absolute percent units. Significant p-values are black, non-significant p-values are grey. N = 500 for (**a–c**) N = 430 for d. Error bars represent 0.95 confidence intervals. CV = Coefficient of variation [calculated as standard deviation/mean]; MCAo = Middle cerebral artery occlusion.

**Figure 3:**
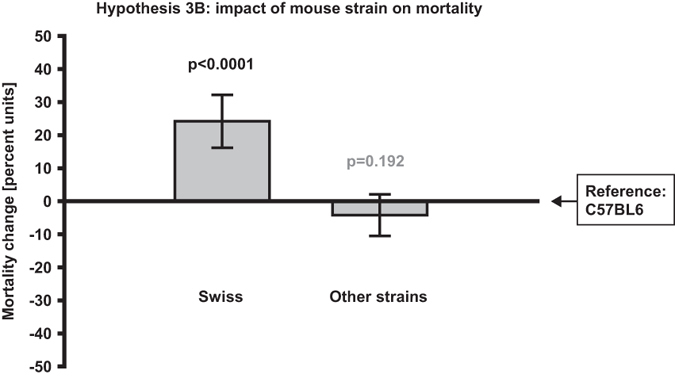
Method parameters’ impact on mortality rate. Swiss strain was found to significantly increase mortality rate compared to the reference C57BL6. The variables *Occlusion duration, Type of middle cerebral artery occlusion procedure* and *Occluding filament type* were removed in the backward exclusion step of the regression model due to small explanatory value and therefore results of hypotheses 1B, 2B and 4B could not be presented. Bars represent change in *Mortality rate,* measured in absolute percent units. Significant p-values are black, non-significant p-values are grey. N = 80. Error bars represent 0.95 confidence intervals.

Regarding the effect of *Occlusion duration* on the outcome *Infarct size coefficient of variation*, only the category *Permanent* turned out to significantly decrease the variability compared to the reference category *Short transient* (−8.6%, CI: −15.3 to −1.9%; p = 0.012; Fig. 2a). No impact of *Occlusion duration* on *Mortality rate* was found (those categories were removed in the backward exclusion step of the statistical analysis and therefore not presented in Fig. 3).

### Impact of type of focal cerebral ischemia procedure on infarct size variability and mortality (hypotheses 2A and 2B)

In the analysis of cerebral ischemia procedures, the *Emboli/clot method* strongly augmented the *Infarct size coefficient of variation* (+25.9, CI: +8.2 to +43.6; p = 0.004; Fig. 2b) in comparison to the reference category *Intraluminal Filament. Mortality rate* was not significantly affected by cerebral ischemia procedure (variables removed during the backward exclusion procedure).

### Impact of mouse strain on infarct size variability and mortality (hypotheses 3A and 3B)

Strain affected both *Infarct size coefficient of variation* and *Mortality rate* significantly. Overall, the majority of the strains seemed to increase the variability compared to the reference category *C57BL6,* with the strongest positive regression coefficient being found for *Mixed C57BL6/129* (+22.8%, CI: +12.5 to 33.1%; p < 0.0001; Fig. 2c) and *129* (+15.9%, CI: +8.3 to 33.1%; p < 0.0001; Fig. 2c). The only strain category that significantly reduced the variability compared to the reference was *Swiss* (−5.7%, CI:−11.2 to −0.3%; p = 0.038; Fig. 2c). Except for the reference, two strain categories were included in the mortality analysis and only *Swiss* had a significant impact by increasing the *Mortality rate* (+24.2%, CI: +16.2 to +32.2%; p < 0.0001; Fig. 3).

### Impact of filament coating type on infarct size variability and mortality (hypotheses 4A and 4B)

In the filament subanalyses, including only articles where the intraluminal filament method had been used, none of the coating type categories (*Occluding filament type*) seemed to affect the infarct size variability. Although the categories remained in the final enter model, the regression coefficients were small (Fig. 2d). Regarding *Mortality rate*, coating categories did not make it through the backward exclusion (hence, they were not significant).

### Background data

**Figure 4:**
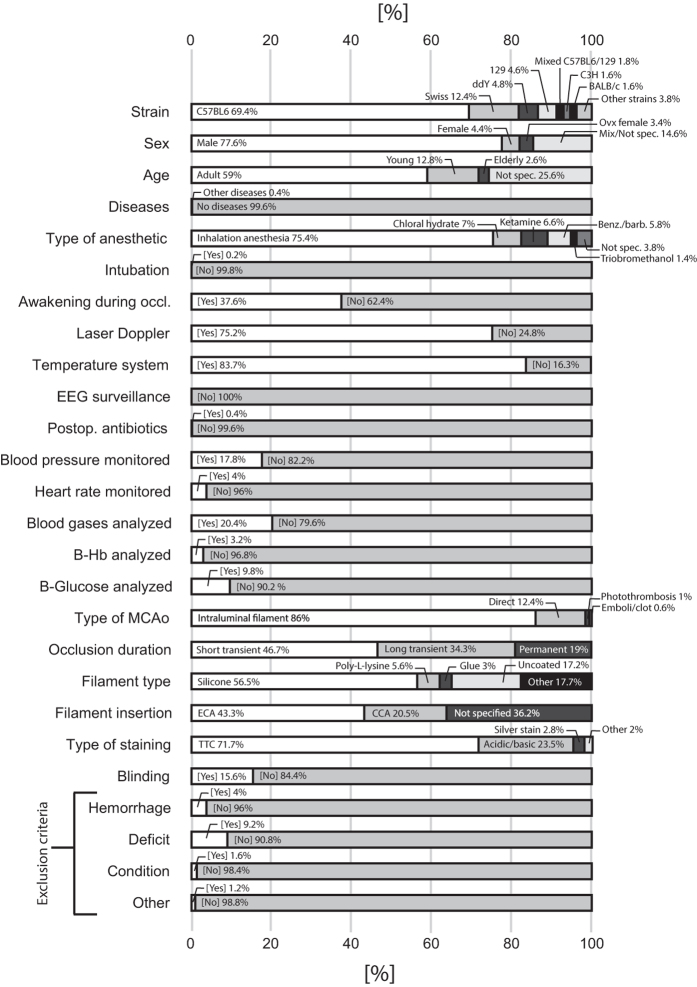
Frequencies of registered categories in the 500 control groups. The figure also includes variables that were omitted from statistical analysis due to too few articles providing these data. Some variable names are abbreviated, see Table 1 for extended descriptions. EEG = Electroencephalography; B = Blood; MCAo = Middle cerebral artery occlusion; TTC = Triphenyl tetrazolium chloride; ECA = External carotid artery; CCA = Common carotid artery.

The *Infarct size coefficient of variation* (in the total 500 control groups^9^,^10^,^11^,^12^,^13^,^14^,^15^,^16^,^17^,^18^,^19^,^20^,^21^,^22^,^23^,^24^,^25^,^26^,^27^,^28^,^29^,^30^,^31^,^32^,^33^,^34^,^35^,^36^,^37^,^38^,^39^,^40^,^41^,^42^,^43^,^44^,^45^,^46^,^47^,^48^,^49^,^50^,^51^,^52^,^53^,^54^,^55^,^56^,^57^,^58^,^59^,^60^,^61^,^62^,^63^,^64^,^65^,^66^,^67^,^68^,^69^,^70^,^71^,^72^,^73^,^74^,^75^,^76^,^77^,^78^,^79^,^80^,^81^,^82^,^83^,^84^,^85^,^86^,^87^,^88^,^89^,^90^,^91^,^92^,^93^,^94^,^95^,^96^,^97^,^98^,^99^,^100^,^101^,^102^,^103^,^104^,^105^,^106^,^107^,^108^,^109^,^110^,^111^,^112^,^113^,^114^,^115^,^116^,^117^,^118^,^119^,^120^,^121^,^122^,^123^,^124^,^125^,^126^,^127^,^128^,^129^,^130^,^131^,^132^,^133^,^134^,^135^,^136^,^137^,^138^,^139^,^140^,^141^,^142^,^143^,^144^,^145^,^146^,^147^,^148^,^149^,^150^,^151^,^152^,^153^,^154^,^155^,^156^,^157^,^158^,^159^,^160^,^161^,^162^,^163^,^164^,^165^,^166^,^167^,^168^,^169^,^170^,^171^,^172^,^173^,^174^,^175^,^176^,^177^,^178^,^179^,^180^,^181^,^182^,^183^,^184^,^185^,^186^,^187^,^188^,^189^,^190^,^191^,^192^,^193^,^194^,^195^,^196^,^197^,^198^,^199^,^200^,^201^,^202^,^203^,^204^,^205^,^206^,^207^,^208^,^209^,^210^,^211^,^212^,^213^,^214^,^215^,^216^,^217^,^218^,^219^,^220^,^221^,^222^,^223^,^224^,^225^,^226^,^227^,^228^,^229^,^230^,^231^,^232^,^233^,^234^,^235^,^236^,^237^,^238^,^239^,^240^,^241^,^242^,^243^,^244^,^245^,^246^,^247^,^248^,^249^,^250^,^251^,^252^,^253^,^254^,^255^,^256^,^257^,^258^,^259^,^260^,^261^,^262^,^263^,^264^,^265^,^266^,^267^,^268^,^269^,^270^,^271^,^272^,^273^,^274^,^275^,^276^,^277^,^278^,^279^,^280^,^281^,^282^,^283^,^284^,^285^,^286^,^287^,^288^,^289^,^290^,^291^,^292^,^293^,^294^,^295^,^296^,^297^,^298^,^299^,^300^,^301^,^302^,^303^,^304^,^305^,^306^,^307^,^308^,^309^,^310^,^311^,^312^,^313^,^314^,^315^,^316^,^317^,^318^,^319^,^320^,^321^,^322^,^323^,^324^,^325^,^326^,^327^,^328^,^329^,^330^,^331^,^332^,^333^,^334^,^335^,^336^,^337^,^338^,^339^,^340^,^341^,^342^) was on average 29.5 ± 19.2% (range 0.9–135.5%) while *Mortality rate* (calculated from the 80 control groups reporting this) was 14 ± 12% (range 0–83%). *Number of animals per group* was on average 8.4 ± 3.1 (range 3–26). The reported body weight group means were on average 25.6 ± 4.0 g (range 18–45). The average time from induction of cerebral ischemia until sacrifice and damage evaluation was 65.0 ± 104.5 h (range 1.5–1008 h), with a median of 24 h. Frequencies of the different categories of the categorical variables are presented in Fig. 4.

## Discussion

The current study shows that the use of *Swiss* and *C57BL6* mice as well as *Permanent* occlusion of the middle cerebral artery renders the lowest infarct size variability. *Emboli/clot* methods, although represented by few control groups, increased variability. Of the methodological factors investigated, only *Swiss* mice was found to have a significant impact on *Mortality rate* by increasing it compared to the reference strain. Effect sizes were large, with many parameters changing the outcomes more than 10% in absolute terms. In addition to the findings pertaining to the hypotheses, several other interesting observations were made, such as the beneficial effects of *Laser Doppler surveillance* on *Infarct size coefficient of variation* and that *Mortality rate* was higher with *Elderly* mice. However, since this study was designed as a hypothesis-driven meta-analysis, results not related to the factor-outcome relations stated a priori should be interpreted with caution and considered merely hypothesis-generating (nevertheless, all findings are presented in Tables S1, S2, S3 and S4 in the Supplementary for readers with special interest in certain methodological parameters).

As mentioned above, comparing all possible combinations of methodological factors experimentally would be a tedious endeavor. However, there are example of studies that investigated one or a few parameters in order to optimize the ischemia model. The majority of these focused on different mouse strains and they did not specifically present or statically compare effect on outcome variability. However, the coefficients of variation can be calculated from mean infarct size and standard deviation similarly to what was done for the regression model in the current meta-analysis. In line with our findings, 129 mice tended to have smaller infarcts with larger infarct size variation compared to C57BL6^343^,^344^,^345^,^346^, although the extent of difference varied. Not corroborated by the current meta-analysis, two of these studies also included BALB/c in the comparison and found that this strain produced infarcts even bigger than those of C57BL6 but with smaller coefficient of variation^344^,^345^. One of the studies presented mortality and concluded that BALB/c had the highest rate, C57BL6 the lowest and 129 was in between the other two strains^344^. We found an increased mortality with the Swiss strain, but only two other categories were represented in that analysis, C57BL6 and other strains.

A few previous articles describe the effects of different middle cerebral artery occlusion durations but the results are discordant. Similar to what we found, both Tsuchia *et al.*^347^ and Mao *et al.*^348^ reported lower coefficients of variation for permanent occlusion compared to transient while in another study, the results were the other way around^343^. In a study with occlusion durations corresponding to our categories *Short transient* (up to 60 min) and *Long transient* (>60 min), short transient occlusion was more favorable in terms of infarct size variability. Regarding mortality rate, similar inconsistency was found with one study presenting lower values for transient occlusion^347^, and one for permanent^349^.

Proper comparisons between methods for ischemia induction in mice are lacking in the literature. This lack is probably explained by the high cost of introducing a new MCAo method in a laboratory, emphasizing the importance of meta-analyses like the current as an alternative. One study looked at the effect of Poly-L-lysine but, like us, found no effect^350^. Filament coating length^351^,^352^ and filament size^347^,^353^ has been investigated but these parameters were not included in our study due to poor reporting in the included articles.

When comparing the current study with the previous rat meta-analysis (described above), some aspect are worth commenting. Similar to what was described herein, emboli methods were found to render larger coefficient of variation of the infarct size than filament, direct and photothrombosis methods^8^. However, infarcts induced by endothelin (not represented in the current mice analysis) were even more inconsistent. Further, although not included in the main hypotheses of the rat study, permanent ischemia had the lowest variability when comparing different occlusion durations both for rats and mice^8^. The rat and mice studies also differ regarding some parameters. For example, no significant differences were found for mice between types of coatings in the filament subanalysis, whereas silicone decreased variability for rats^8^.

The main problem with high infarct size variability is the resulting lack of statistical power if the sample sizes are not adjusted accordingly, which has been discussed in several reviews^5^,^354^,^355^. Statistical power (1-β) is often discussed in relation to negative findings, e.g. to evaluate if a study was adequately designed to detect a treatment effect of a substance and hence if the negative results are to trust or not. However, statistical power is of importance also for studies with positive findings (i.e. when a treatment effect is found)^5^,^356^. Low statistical power is associated to the publication bias phenomenon since negative findings are generally less likely to be published, which can distort interpretation of meta-analyses^357^. To support the claim that statistical power in experimental stroke studies is often low, the average power of the studies included in current meta-analysis can be calculated based on the extracted data: The average group sizes were 8.4 and the average coefficient of variation for infarct sizes 29.5%, which at a significance level of 0.05 gives a power of 59% to detect a 30% difference between groups (calculation based on parametric comparison between two-groups, for more three groups or more and non-parametric methods, the number would be even lower). Ethical boards demanding researchers to minimize number of animals (the three Rs principle^7^) might explain why too small group sizes are often used, but economic as well as practical aspects are also likely to contribute. Lack of adequate statistical training or no available statistician to consult regarding these issues should also be mentioned as an option. So in addition to optimizing the model to produce consistent lesions and minimize mortality, it is important to perform a priori power calculations in order to avoid the abovementioned problems.

The issue of mortality is somewhat related to outcome variability and power calculations in that higher mortality require larger group sizes to attain sufficient power. However, there is another side to the problem as well. Regarding the statistical analysis, it is not uncomplicated to incorporate mortality in the standard parametric methods which might explain why this information in most cases is not even mentioned. A non-parametric approach, with mortality included as worst possible outcome, is an option that has been utilized in our laboratory^358^,^359^ but either way, the absolute minimum should be to report these data. The risk when omitting mortality rate data can be illustrated by the possible scenario of a toxic substance that seems to decrease infarct sizes compared to a placebo group, only because all mice with large infarcts in the treatment group died. In the current meta-analysis, it might seem surprising that the effects on mortality were generally moderate (e.g. no significant effect of occlusion time). However, mortality data was only available for 80/500 control groups. A low number of observations weakens a regression model with many predictor variables, and this should be considered when conclusions are drawn.

The main strength of the present meta-analysis is the large number of articles included, and that the effects of many methodological factors are investigated together in one single statistical model. However, this approach is relatively novel, warranting a discussion about some aspects of the design:

 - The impact of each control group were weighed according to number of animals which might be problematic when analyzing coefficient of variation, since researchers knowing that they have large variability in their model probably compensate by including more animals.

 - The effect of publication bias has to be considered, as studies with large coefficients of variation might produce negative results that are more likely to remain unpublished.

 - Although many possible confounders were recorded and controlled for, accounting for all details of the included experiments is beyond the reach of even a meta-analytical approach. Impact of different vendors and skill of the surgeon are just a couple of factors that could not be assessed. For mathematical reasons, categories have also, as described in the Methods section, been reduced to larger categories, meaning that differences within categories may be lost.

 - 500 control groups are included but only 334 articles, meaning that several articles contributed with more than one control group. It is not strictly statistically appropriate to analyze these independently but creating categories for all unique studies would have made the statistical analysis impossible.

In conclusion, the methodological choices are of major importance for consistent results and advantageous animal models. However, although it may be relevant to adjust the experimental setup to minimize infarct size variability and mortality rate, other important components such as similarity to the clinical situation have to be taken into consideration. For this reason, it might be motivated in some studies to use the emboli method or elderly animals even though this might increase the outcome variability and mortality, respectively. In either case, the current study enables a more precise estimation of variability and mortality a priori given a certain experimental setup, thereby facilitating proper power calculations.

## Methods

### Overview

The basic outline for the study was pre-defined and consisted of the following steps:

1. Variables to be studied were chosen.
2. Data about chosen variables were extracted from relevant articles.
3. Variable categories were refined based on extraction results.
4. Statistical analyses were performed on variables left after refinement.

### Article inclusion

Relevant articles were identified in the Medline database via PubMed using the search string (mcao or “middle cerebral artery occlusion” or “MCA occlusion” or “stroke” or “cerebral ischemia” or “brain ischemia”) and (mouse or mice), resulting in over 6,000 hits. The articles were consecutively assessed for inclusion, in order of PubMed identifier, starting with the most recent article January 9^th^ 2012. The inclusion criteria were:

- Article written in English.
- Original research article.
- Experiments performed using living mice.
- Mice inflicted one single focal cerebral ischemic lesion.
- Infarct sizes measured and results presented.
- Inclusion of a control group, untreated except for vehicle/placebo treatment.
- Experiment adequately described.

### Data extraction

Control group data were extracted from all included articles. If an article described more than one control group, differing in any methodological aspect, these were included separately and analyzed independently. The principle “if it was not described, it was not performed” was adhered to throughout the process. Methodological factors to be extracted were chosen based on our previous rat meta-analysis^8^ and personal experience. See Table 1 for a complete list of all variables that we intended to extract. The goal was to gather as much relevant data as possible in order to build a good statistical model.

To perform a proper power calculation for such a large multiple regression model is a very complex task. Instead, the sample size estimation was based on our previous meta-analysis with a similar design. Furthermore, we performed interim saturation analyses after 400 and 450 included control groups to check when the results had stabilized, i.e. no changes in overall trends occurred. In total, 500 control groups from 334 articles (see Supplementary methods for a complete list of references) were included and 1784 articles were excluded (Fig. 1).

### Processing of data

#### Category refinement

To avoid small categories being attributed statistically unsubstantiated explanatory value, categories represented by less than 5 control groups were pooled in an *Other* category for that specific variable. The overall effects on the two outcome variables (*Infarct size variation* and *Mortality rate*; hypothesis 1A, 1B, 2A, 2B, 3A and 3B) were tested in two independent models and in addition, the filament method subanalysis (hypotheses 4A and 4B) had to be performed separately. Each of the resulting four regression models comprised different numbers of control groups since not all articles reported on mortality and obviously only studies using the intraluminal filament model could be included for the filament subanalysis. Hence, in some cases a category represented by more than 5 control groups in one regression model was reduced to less than 5 groups in another and thus incorporated in the *Others* category, in line with the general category size principle described above. See Supplementary methods for a detailed description of processing of data. Also, in Tables S1, S2, S3 and S4 (Supplementary) the final categories for each regression model are presented.

#### Excluded variables

The following variables were originally intended to be incorporated into the model, but since none or very few articles reported these data they had to be omitted: *Diseases, Intubation, EEG supervision, Postoperative antibiotics, Filament tip diameter, Filament coating length and Exclusion rate.*

#### Statistics

As described above, eight main hypotheses were stated a priori:

1. Middle cerebral artery occlusion duration affects (A) infarct size variability and (B) mortality.
2. Type of focal cerebral ischemia procedure affects (A) infarct size variability and (B) mortality.
3. Mouse strain affects (A) infarct size variability and (B) mortality.
4. In studies using the intraluminal filament method, the type of filament affects (A) infarct size variability and (B) mortality.

Since large multiple regression models may suggest a wide range of unexpected associations between variables, a limited set of predefined hypotheses were established to lower the risk of finding falsely significant results due to multiple comparisons (type I errors). Findings not related to these were interpreted with caution and considered merely hypothesis-generating. Due to the risk of type II-errors, corrections for multiple comparisons were not performed.

All categories were dummy-converted before analysis (Table 1). For binomial variables, lack of a specific methodological factor, i.e. [No], was considered the reference category whereas the most common category was chosen as baseline for variables with more than two categories. The data were analyzed using weighted multiple linear regression in two steps. First, a backward exclusion procedure identified factors that contributed significantly to the model and removed the rest. Subsequently, an enter model was performed, in which significant factors identified was manually complemented by lacking dummy variables that were excluded in the previous step (presented in Table S1, S2, S3 and S4). Weighing of cases was performed according to the number of animals in each control group; hence, groups with more animals had larger impact on the statistical model than groups with few animals. Based on the hypotheses, four regression models (one for hypotheses 1A, 2A and 3A; one for hypotheses 1B, 2B and 3B; one for hypothesis 4A and one for hypothesis 4B) were built to test the combined effects of all factors on the two separate outcome measures, *Infarct size coefficient of variation* or *Mortality*. In this way, when investigating one of the specific hypotheses, the model controlled for the other predictor variables. The models passed residual checks and multicollinearity tests. All statistical calculations were performed in SPSS (Version 23, IBM Corporation, Armonk, NY, USA). P-values <0.05 were considered significant. Regarding results from the meta-analysis, 95% confidence interval were provided, otherwise data were presented as mean ± standard deviation.

## Additional Information

**How to cite this article**: Ingberg, E. *et al.* Method parameters' impact on mortality and variability in mouse stroke experiments: a meta-analysis. *Sci. Rep.* **6**, 21086; doi: 10.1038/srep21086 (2016).

## Supplementary Material

Supplementary Information

## Figures and Tables

**Table 1:** Extracted factors and outcome measures.

Factor/outcome measure	Data type	Final categories or unit*	Reference category for regression model
Mouse property factors			
Strain	Category	I. C57BL6	C57BL6
		II. Swiss	
		III. ddY	
		IV. 129	
		V. Mixed C57BL6/129	
		VI. C3H	
		VII. BALB/c	
		VIII. Other strains	
Sex	Category	I. Male	Male
		II. Female	
		III. Ovx female	
		IV. Other sex	
Age	Category	I. Adult (>2, <12 months)	Adult
		II. Young (0–2 months)	
		III. Elderly (≥12 months)	
		IV. Age not specified	
Weight	Continuous	Grams	NA
Diseases**	Category	I. Other diseases	NA
Anesthesia factors			
Type of anesthetic	Category	I. Inhalation anesthesia	Inhalation anesthesia
		II. Chloral hydrate	
		III. Ketamine	
		IV. Benzodiazepines and barbiturates	
		V. Tribromoethanol	
		VI. Anesthetic not specified	
Intubation**	Category, Binomial	[No]	[No]
		[Yes]	
Awakening during occlusion	Category, Binomial	[No]	[No]
		[Yes]	
Laser Doppler surveillance	Category, Binomial	[No]	[No]
		[Yes]	
Temperature feedback system	Category, Binomial	[No]	[No]
		[Yes]	
Electroencephalographic surveillance**	Category, Binomial	[No]	[No]
		[Yes]	
Postoperative antibiotics**	Category, Binomial	[No]	[No]
		[Yes]	
Blood pressure monitored	Category, Binomial	[No]	[No]
		[Yes]	
Heart rate monitored	Category, Binomial	[No]	[No]
		[Yes]	
Blood gases/O_2_ saturation analyzed	Category, Binomial	[No]	[No]
		[Yes]	
Blood hemoglobin analyzed	Category, Binomial	[No]	[No]
		[Yes]	
Blood glucose analyzed	Category, Binomial	[No]	[No]
		[Yes]	
Focal ischemia procedure factors			
Type of middle cerebral artery occlusion procedure	Category	I. Intraluminal filament	Intraluminal filament
		II. Direct, mechanical	
		III. Photothrombosis	
		IV. Emboli/clot	
Occlusion duration	Category	I. Short transient (up to 60 minutes)	Short transient (up to 60 minutes)
		II. Long transient (>60 minutes)	
		III. Permanent	
Occluding filament type (only studies using the intraluminal filament method)	Category	I. Silicone	Silicone
		II. Poly-L-lysine	
		III. Glue	
		IV. Uncoated	
		V. Other coatings	
Filament coating length (only studies using the intraluminal filament method)**	Continuous	Millimeters	NA
Filament tip diameter**	Continuous	Millimeter	NA
Filament insertion (only using the intraluminal filament method)	Category	I. External carotid artery	External carotid artery
		II. Common carotid artery	
		III. Vessel not specified	
Analysis procedure factors			
Time after ischemia for evaluation of damage	Continuous	Hours	NA
Type of staining	Category	I. Triphenyl tetrazolium chloride (TTC)	Triphenyl tetrazolium chloride (TTC)
		II. Acidic/basic stain	
		III. Silver stain	
		IV. Other stains	
Blinding of infarct size measurement procedure	Category, Binomial	[No]	[No]
		[Yes]	
Exclusion based on hemorrhage	Category, Binomial	[No]	[No]
		[Yes]	
Exclusion based on neurological deficit score	Category, Binomial	[No]	[No]
		[Yes]	
Exclusion based on bad clinical condition	Category, Binomial	[No]	[No]
		[Yes]	
Exclusion based on other criteria	Category, Binomial	[No]	[No]
		[Yes]	
Outcome measures			
Infarct size coefficient of variation	Continuous	%	NA
Mortality rate	Continuous	%	NA

*Only categories represented by at least 5 control groups were included in the analysis to avoid statistically inadequate attribution of explanatory value to too small categories. Categories represented by less than 5 control groups were in the analysis included in an Others category. For the same reason, some categories presented here were merged with the Others category for hypotheses 1B, 2B, 3B, 4A and 4B (see Supplementary methods and Tables S2, S3 and S4). Further, some other reductions in number of categories were performed, described in detail in Supplementary methods.

**Too few articles reported on this variable; omitted from analysis.

## References

[b1] Donnan G. A., Fisher M., Macleod M. & Davis S. M. Stroke. Lancet 371, 1612–1623, doi: 10.1016/S0140-6736(08)60694-7 (2008). 18468545

[b2] Dirnagl U. & Endres M. Found in translation: preclinical stroke research predicts human pathophysiology, clinical phenotypes, and therapeutic outcomes. Stroke 45, 1510–1518, doi: 10.1161/STROKEAHA.113.004075 (2014). 24652307

[b3] O’Collins V. E. *et al.* 1,026 experimental treatments in acute stroke. Ann Neurol 59, 467–477, doi: 10.1002/ana.20741 (2006). 16453316

[b4] Endres M. *et al.* Improving outcome after stroke: overcoming the translational roadblock. Cerebrovasc Dis 25, 268–278, doi: 10.1159/000118039 (2008). 18292653

[b5] Dirnagl U. Bench to bedside: the quest for quality in experimental stroke research. J Cereb Blood Flow Metab 26, 1465–1478, doi: 10.1038/sj.jcbfm.9600298 (2006). 16525413

[b6] Macleod M. R. *et al.* Evidence for the efficacy of NXY-059 in experimental focal cerebral ischaemia is confounded by study quality. Stroke 39, 2824–2829, doi: 10.1161/STROKEAHA.108.515957 (2008). 18635842

[b7] Russel W. & Burch R. The principles of humane experimental technique. *Universities Federation for Animal Welfare, Wheathamstead, England* (1959 (as reprinted 1992)).

[b8] Strom J. O., Ingberg E., Theodorsson A. & Theodorsson E. Method parameters’ impact on mortality and variability in rat stroke experiments: a meta-analysis. BMC Neurosci 14, 41, doi: 10.1186/1471-2202-14-41 (2013). 23548160 PMC3637133

[b9] Moussaieff A. *et al.* Protective effects of incensole acetate on cerebral ischemic injury. Brain Res 1443, 89–97, doi: 10.1016/j.brainres.2012.01.001 (2012). 22284622 PMC3294134

[b10] Wang B. *et al.* Histone deacetylase inhibition activates transcription factor Nrf2 and protects against cerebral ischemic damage. Free Radic Biol Med 52, 928–936, doi: 10.1016/j.freeradbiomed.2011.12.006 (2012). 22226832 PMC6010182

[b11] Zheng C. *et al.* NAD(+) administration decreases ischemic brain damage partially by blocking autophagy in a mouse model of brain ischemia. Neurosci Lett 512, 67–71, doi: 10.1016/j.neulet.2012.01.007 (2012). 22260797

[b12] Harada S. *et al.* Honokiol suppresses the development of post-ischemic glucose intolerance and neuronal damage in mice. J Nat Med 66, 591–599, doi: 10.1007/s11418-011-0623-x (2012). 22261858

[b13] Lu H. *et al.* Netrin-1 hyperexpression in mouse brain promotes angiogenesis and long-term neurological recovery after transient focal ischemia. Stroke 43, 838–843, doi: 10.1161/STROKEAHA.111.635235 (2012). 22223243

[b14] Fujioka M. *et al.* ADAMTS13 gene deletion enhances plasma high-mobility group box1 elevation and neuroinflammation in brain ischemia-reperfusion injury. Neurol Sci 33, 1107–1115, doi: 10.1007/s10072-011-0913-9 (2012). 22212812

[b15] Park J. S. *et al.* Anti-inflammatory mechanism of compound K in activated microglia and its neuroprotective effect on experimental stroke in mice. J Pharmacol Exp Ther 341, 59–67, doi: 10.1124/jpet.111.189035 (2012). 22207656

[b16] Elvington A. *et al.* Pathogenic natural antibodies propagate cerebral injury following ischemic stroke in mice. J Immunol 188, 1460–1468, doi: 10.4049/jimmunol.1102132 (2012). 22198950 PMC3262954

[b17] Colak G. & Johnson G. V. Complete transglutaminase 2 ablation results in reduced stroke volumes and astrocytes that exhibit increased survival in response to ischemia. Neurobiol Dis 45, 1042–1050, doi: 10.1016/j.nbd.2011.12.023 (2012). 22198379 PMC3276707

[b18] Harada S., Fujita-Hamabe W. & Tokuyama S. Ameliorating effect of hypothalamic brain-derived neurotrophic factor against impaired glucose metabolism after cerebral ischemic stress in mice. J Pharmacol Sci 118, 109–116 (2012). 22198007 10.1254/jphs.11164fp

[b19] Liang J. *et al.* Participation of MCP-induced protein 1 in lipopolysaccharide preconditioning-induced ischemic stroke tolerance by regulating the expression of proinflammatory cytokines. J Neuroinflammation 8, 182, doi: 10.1186/1742-2094-8-182 (2011). 22196138 PMC3260209

[b20] Shang J., Liu N., Tanaka N. & Abe K. Expressions of hypoxic stress sensor proteins after transient cerebral ischemia in mice. J Neurosci Res 90, 648–655, doi: 10.1002/jnr.22776 (2012). 22183753

[b21] Hoda M. N. *et al.* Sex-independent neuroprotection with minocycline after experimental thromboembolic stroke. Exp Transl Stroke Med 3, 16, doi: 10.1186/2040-7378-3-16 (2011). 22177314 PMC3287111

[b22] Hase Y. *et al.* Cilostazol, a phosphodiesterase inhibitor, prevents no-reflow and hemorrhage in mice with focal cerebral ischemia. Exp Neurol 233, 523–533, doi: 10.1016/j.expneurol.2011.11.038 (2012). 22173318

[b23] Kim G. S. *et al.* Release of mitochondrial apoptogenic factors and cell death are mediated by CK2 and NADPH oxidase. J Cereb Blood Flow Metab 32, 720–730, doi: 10.1038/jcbfm.2011.176 (2012). 22146192 PMC3318149

[b24] Venna V. R., Li J., Benashski S. E., Tarabishy S. & McCullough L. D. Preconditioning induces sustained neuroprotection by downregulation of adenosine 5’-monophosphate-activated protein kinase. Neuroscience 201, 280–287, doi: 10.1016/j.neuroscience.2011.11.014 (2012). 22120436 PMC3258333

[b25] Masuko T. *et al.* Antagonism of NMDA receptors by butanesulfonyl-homospermine guanidine and neuroprotective effects in *in vitro* and *in vivo*. Neurosci Lett 506, 251–255, doi: 10.1016/j.neulet.2011.11.017 (2012). 22119002

[b26] Denes A., Ferenczi S. & Kovacs K. J. Systemic inflammatory challenges compromise survival after experimental stroke via augmenting brain inflammation, blood- brain barrier damage and brain oedema independently of infarct size. J Neuroinflammation 8, 164, doi: 10.1186/1742-2094-8-164 (2011). 22114895 PMC3235982

[b27] De Silva T. M., Brait V. H., Drummond G. R., Sobey C. G. & Miller A. A. Nox2 oxidase activity accounts for the oxidative stress and vasomotor dysfunction in mouse cerebral arteries following ischemic stroke. PLoS One 6, e28393, doi: 10.1371/journal.pone.0028393 (2011). 22164282 PMC3229592

[b28] Nagai M. *et al.* Role of blood cell-associated angiotensin II type 1 receptors in the cerebral microvascular response to ischemic stroke during angiotensin-induced hypertension. Exp Transl Stroke Med 3, 15, doi: 10.1186/2040-7378-3-15 (2011). 22087550 PMC3240825

[b29] Cipriani R. *et al.* CX3CL1 is neuroprotective in permanent focal cerebral ischemia in rodents. J Neurosci 31, 16327–16335, doi: 10.1523/JNEUROSCI.3611-11.2011 (2011). 22072684 PMC6633249

[b30] Ogle M. E., Gu X., Espinera A. R. & Wei L. Inhibition of prolyl hydroxylases by dimethyloxaloylglycine after stroke reduces ischemic brain injury and requires hypoxia inducible factor-1alpha. Neurobiol Dis 45, 733–742, doi: 10.1016/j.nbd.2011.10.020 (2012). 22061780 PMC3286647

[b31] Liu F., Benashski S. E., Xu Y., Siegel M. & McCullough L. D. Effects of chronic and acute oestrogen replacement therapy in aged animals after experimental stroke. J Neuroendocrinol 24, 319–330, doi: 10.1111/j.1365-2826.2011.02248.x (2012). 22053957 PMC3580836

[b32] Pleines I. *et al.* Megakaryocyte-specific RhoA deficiency causes macrothrombocytopenia and defective platelet activation in hemostasis and thrombosis. Blood 119, 1054–1063, doi: 10.1182/blood-2011-08-372193 (2012). 22045984

[b33] Strecker J. K. *et al.* Monocyte chemoattractant protein-1-deficiency impairs the expression of IL-6, IL-1beta and G-CSF after transient focal ischemia in mice. PLoS One 6, e25863, doi: 10.1371/journal.pone.0025863 (2011). 22031820 PMC3198727

[b34] Sieber M. W., Claus R. A., Witte O. W. & Frahm C. Attenuated inflammatory response in aged mice brains following stroke. PLoS One 6, e26288, doi: 10.1371/journal.pone.0026288 (2011). 22028848 PMC3196544

[b35] Tang X. N., Zheng Z., Giffard R. G. & Yenari M. A. Significance of marrow-derived nicotinamide adenine dinucleotide phosphate oxidase in experimental ischemic stroke. Ann Neurol 70, 606–615, doi: 10.1002/ana.22476 (2011). 22028221 PMC3205431

[b36] Li S. Y. *et al.* Lutein enhances survival and reduces neuronal damage in a mouse model of ischemic stroke. Neurobiol Dis 45, 624–632, doi: 10.1016/j.nbd.2011.10.008 (2012). 22024715

[b37] Vartanian K. B. *et al.* LPS preconditioning redirects TLR signaling following stroke: TRIF-IRF3 plays a seminal role in mediating tolerance to ischemic injury. J Neuroinflammation 8, 140, doi: 10.1186/1742-2094-8-140 (2011). 21999375 PMC3217906

[b38] Ren X. *et al.* Myelin specific cells infiltrate MCAO lesions and exacerbate stroke severity. Metab Brain Dis 27, 7–15, doi: 10.1007/s11011-011-9267-5 (2012). 21989743 PMC3270145

[b39] Yung L. M. *et al.* Sphingosine kinase 2 mediates cerebral preconditioning and protects the mouse brain against ischemic injury. Stroke 43, 199–204, doi: 10.1161/STROKEAHA.111.626911 (2012). 21980199 PMC3246529

[b40] Rodrigues S. F. & Granger D. N. Cerebral microvascular inflammation in DOCA salt-induced hypertension: role of angiotensin II and mitochondrial superoxide. J Cereb Blood Flow Metab 32, 368–375, doi: 10.1038/jcbfm.2011.139 (2012). 21971354 PMC3272604

[b41] Zhang J. Y. *et al.* Leptin attenuates cerebral ischemia/reperfusion injury partially by CGRP expression. Eur J Pharmacol 671, 61–69, doi: 10.1016/j.ejphar.2011.09.170 (2011). 21968137

[b42] Liang X. *et al.* Signaling via the prostaglandin E(2) receptor EP4 exerts neuronal and vascular protection in a mouse model of cerebral ischemia. J Clin Invest 121, 4362–4371, doi: 10.1172/JCI46279 (2011). 21965326 PMC3204834

[b43] Akiyoshi K. *et al.* Recombinant T cell receptor ligands improve outcome after experimental cerebral ischemia. Transl Stroke Res 2, 404–410, doi: 10.1007/s12975-011-0085-1 (2011). 21961027 PMC3181103

[b44] Morancho A. *et al.* A new method for focal transient cerebral ischaemia by distal compression of the middle cerebral artery. Neuropathol Appl Neurobiol 38, 617–627, doi: 10.1111/j.1365-2990.2012.01252.x (2012). 22289071

[b45] Wang P., Tian W. W., Song J., Guan Y. F. & Miao C. Y. Deficiency of NG^2+^ cells contributes to the susceptibility of stroke-prone spontaneously hypertensive rats. CNS Neurosci Ther 17, 327–332, doi: 10.1111/j.1755-5949.2011.00265.x (2011). 21951366 PMC6493835

[b46] Texel S. J. *et al.* Ceruloplasmin deficiency reduces levels of iron and BDNF in the cortex and striatum of young mice and increases their vulnerability to stroke. PLoS One 6, e25077, doi: 10.1371/journal.pone.0025077 (2011). 21949858 PMC3174999

[b47] Jung J. E., Kim G. S. & Chan P. H. Neuroprotection by interleukin-6 is mediated by signal transducer and activator of transcription 3 and antioxidative signaling in ischemic stroke. Stroke 42, 3574–3579, doi: 10.1161/STROKEAHA.111.626648 (2011). 21940958 PMC3395465

[b48] Chang C. C. *et al.* Prodigiosin inhibits gp91(phox) and iNOS expression to protect mice against the oxidative/nitrosative brain injury induced by hypoxia-ischemia. Toxicol Appl Pharmacol 257, 137–147, doi: 10.1016/j.taap.2011.08.027 (2011). 21925195

[b49] Balkaya M. *et al.* Stress worsens endothelial function and ischemic stroke via glucocorticoids. Stroke 42, 3258–3264, doi: 10.1161/STROKEAHA.110.607705 (2011). 21921276

[b50] Turtzo L. C., Siegel C. & McCullough L. D. X chromosome dosage and the response to cerebral ischemia. J Neurosci 31, 13255–13259, doi: 10.1523/JNEUROSCI.0621-11.2011 (2011). 21917808 PMC3179179

[b51] De Meyer S. F., Schwarz T., Schatzberg D. & Wagner D. D. Platelet glycoprotein Ibalpha is an important mediator of ischemic stroke in mice. Exp Transl Stroke Med 3, 9, doi: 10.1186/2040-7378-3-9 (2011). 21914206 PMC3180255

[b52] Kilic U. *et al.* Evidence that membrane-bound G protein-coupled melatonin receptors MT1 and MT2 are not involved in the neuroprotective effects of melatonin in focal cerebral ischemia. J Pineal Res 52, 228–235, doi: 10.1111/j.1600-079X.2011.00932.x (2012). 21913972

[b53] Pham V. *et al.* Insulin-regulated aminopeptidase deficiency provides protection against ischemic stroke in mice. J Neurotrauma 29, 1243–1248, doi: 10.1089/neu.2011.1824 (2012). 21895534

[b54] Shu L. *et al.* Inhibition of neuron-specific CREB dephosphorylation is involved in propofol and ketamine-induced neuroprotection against cerebral ischemic injuries of mice. Neurochem Res 37, 49–58, doi: 10.1007/s11064-011-0582-3 (2012). 21892690

[b55] Pfeilschifter W. *et al.* Activation of sphingosine kinase 2 is an endogenous protective mechanism in cerebral ischemia. Biochem Biophys Res Commun 413, 212–217, doi: 10.1016/j.bbrc.2011.08.070 (2011). 21872577

[b56] Wang B., Cao W., Biswal S. & Dore S. Carbon monoxide-activated Nrf2 pathway leads to protection against permanent focal cerebral ischemia. Stroke 42, 2605–2610, doi: 10.1161/STROKEAHA.110.607101 (2011). 21852618 PMC3278075

[b57] Nakka V. P. *et al.* Increased cerebral protein ISGylation after focal ischemia is neuroprotective. J Cereb Blood Flow Metab 31, 2375–2384, doi: 10.1038/jcbfm.2011.103 (2011). 21847135 PMC3323186

[b58] Ma Y., Mehta S. L., Lu B. & Li P. A. Deficiency in the inner mitochondrial membrane peptidase 2-like (Immp21) gene increases ischemic brain damage and impairs mitochondrial function. Neurobiol Dis 44, 270–276, doi: 10.1016/j.nbd.2011.06.019 (2011). 21824519 PMC3185146

[b59] Kim H. W., Cho K. J., Lee S. K. & Kim G. W. Apoptosis signal-regulating kinase 1 (Ask1) targeted small interfering RNA on ischemic neuronal cell death. Brain Res 1412, 73–78, doi: 10.1016/j.brainres.2011.07.018 (2011). 21803338

[b60] Niu F. N. *et al.* Targeted mutation of Fas ligand gene attenuates brain inflammation in experimental stroke. Brain Behav Immun 26, 61–71, doi: 10.1016/j.bbi.2011.07.235 (2012). 21802508

[b61] Shi Y., Chanana V., Watters J. J., Ferrazzano P. & Sun D. Role of sodium/hydrogen exchanger isoform 1 in microglial activation and proinflammatory responses in ischemic brains. J Neurochem 119, 124–135, doi: 10.1111/j.1471-4159.2011.07403.x (2011). 21797866 PMC3192493

[b62] Brede M. *et al.* alpha(2)-adrenoceptors do not mediate neuroprotection in acute ischemic stroke in mice. J Cereb Blood Flow Metab 31, e1–7, doi: 10.1038/jcbfm.2011.110 (2011). 21792243 PMC3208146

[b63] Wang H. W. *et al.* Deciphering the neuroprotective mechanisms of Bu-yang Huan-wu decoction by an integrative neurofunctional and genomic approach in ischemic stroke mice. J Ethnopharmacol 138, 22–33, doi: 10.1016/j.jep.2011.06.033 (2011). 21784143

[b64] Doeppner T. R. *et al.* Enhancement of endogenous neurogenesis in ephrin-B3 deficient mice after transient focal cerebral ischemia. Acta Neuropathol 122, 429–442, doi: 10.1007/s00401-011-0856-5 (2011). 21779764 PMC3291816

[b65] Iwanami J. *et al.* Effect of angiotensin II type 2 receptor deletion in hematopoietic cells on brain ischemia-reperfusion injury. Hypertension 58, 404–409, doi: 10.1161/HYPERTENSIONAHA.111.177873 (2011). 21768524

[b66] ElAli A. & Hermann D. M. Liver X receptor activation enhances blood-brain barrier integrity in the ischemic brain and increases the abundance of ATP-binding cassette transporters ABCB1 and ABCC1 on brain capillary cells. Brain Pathol 22, 175–187, doi: 10.1111/j.1750-3639.2011.00517.x (2012). 21767321 PMC8029407

[b67] Ren X., Akiyoshi K., Vandenbark A. A., Hurn P. D. & Offner H. Programmed death-1 pathway limits central nervous system inflammation and neurologic deficits in murine experimental stroke. Stroke 42, 2578–2583, doi: 10.1161/STROKEAHA.111.613182 (2011). 21737801 PMC3164218

[b68] Jin K., Xie L., Sun F., Mao X. & Greenberg D. A. Corpus callosum and experimental stroke: studies in callosotomized rats and acallosal mice. Stroke 42, 2584–2588, doi: 10.1161/STROKEAHA.111.613349 (2011). 21737800 PMC3164743

[b69] Park H. A. *et al.* Natural vitamin E alpha-tocotrienol protects against ischemic stroke by induction of multidrug resistance-associated protein 1. Stroke 42, 2308–2314, doi: 10.1161/STROKEAHA.110.608547 (2011). 21719775 PMC3362046

[b70] Lu C. *et al.* TLR2 ligand induces protection against cerebral ischemia/reperfusion injury via activation of phosphoinositide 3-kinase/Akt signaling. J Immunol 187, 1458–1466, doi: 10.4049/jimmunol.1003428 (2011). 21709150 PMC3140609

[b71] Gibson C. L., Coomber B. & Murphy S. P. Progesterone is neuroprotective following cerebral ischaemia in reproductively ageing female mice. Brain 134, 2125–2133, doi: 10.1093/brain/awr132 (2011). 21705427

[b72] Liesz A. *et al.* FTY720 reduces post-ischemic brain lymphocyte influx but does not improve outcome in permanent murine cerebral ischemia. PLoS One 6, e21312, doi: 10.1371/journal.pone.0021312 (2011). 21701599 PMC3119049

[b73] Ye R. *et al.* Ginsenoside Rd attenuates redox imbalance and improves stroke outcome after focal cerebral ischemia in aged mice. Neuropharmacology 61, 815–824, doi: 10.1016/j.neuropharm.2011.05.029 (2011). 21664366

[b74] Wang Z. *et al.* Fusion of core pathways reveals a horizontal synergistic mechanism underlying combination therapy. Eur J Pharmacol 667, 278–286, doi: 10.1016/j.ejphar.2011.05.046 (2011). 21658381

[b75] Ren X. *et al.* Regulatory B cells limit CNS inflammation and neurologic deficits in murine experimental stroke. J Neurosci 31, 8556–8563, doi: 10.1523/JNEUROSCI.1623-11.2011 (2011). 21653859 PMC3111929

[b76] Stevens S. L. *et al.* Multiple preconditioning paradigms converge on interferon regulatory factor-dependent signaling to promote tolerance to ischemic brain injury. J Neurosci 31, 8456–8463, doi: 10.1523/JNEUROSCI.0821-11.2011 (2011). 21653850 PMC3130521

[b77] Min J. *et al.* Neuroprotective effect of cyanidin-3-O-glucoside anthocyanin in mice with focal cerebral ischemia. Neurosci Lett 500, 157–161, doi: 10.1016/j.neulet.2011.05.048 (2011). 21651957

[b78] Su E. J. *et al.* The thrombomodulin analog Solulin promotes reperfusion and reduces infarct volume in a thrombotic stroke model. J Thromb Haemost 9, 1174–1182, doi: 10.1111/j.1538-7836.2011.04269.x (2011). 21645225 PMC3111949

[b79] Shin J. A., Choi J. H., Choi Y. H. & Park E. M. Conserved aquaporin 4 levels associated with reduction of brain edema are mediated by estrogen in the ischemic brain after experimental stroke. Biochim Biophys Acta 1812, 1154–1163, doi: 10.1016/j.bbadis.2011.05.004 (2011). 21641993

[b80] Yamamoto H. *et al.* NDRG4 protein-deficient mice exhibit spatial learning deficits and vulnerabilities to cerebral ischemia. J Biol Chem 286, 26158–26165, doi: 10.1074/jbc.M111.256446 (2011). 21636852 PMC3138246

[b81] Choe C. U. *et al.* CD38 exacerbates focal cytokine production, postischemic inflammation and brain injury after focal cerebral ischemia. PLoS One 6, e19046, doi: 10.1371/journal.pone.0019046 (2011). 21625615 PMC3097994

[b82] Andrabi S. A. *et al.* Iduna protects the brain from glutamate excitotoxicity and stroke by interfering with poly(ADP-ribose) polymer-induced cell death. Nat Med 17, 692–699, doi: 10.1038/nm.2387 (2011). 21602803 PMC3709257

[b83] Xiong X. *et al.* Increased brain injury and worsened neurological outcome in interleukin-4 knockout mice after transient focal cerebral ischemia. Stroke 42, 2026–2032, doi: 10.1161/STROKEAHA.110.593772 (2011). 21597016 PMC3128567

[b84] Jia J. *et al.* Sex differences in neuroprotection provided by inhibition of TRPM2 channels following experimental stroke. J Cereb Blood Flow Metab 31, 2160–2168, doi: 10.1038/jcbfm.2011.77 (2011). 21587268 PMC3210342

[b85] Tsukuda K. *et al.* Irbesartan attenuates ischemic brain damage by inhibition of MCP-1/CCR2 signaling pathway beyond AT(1) receptor blockade. Biochem Biophys Res Commun 409, 275–279, doi: 10.1016/j.bbrc.2011.04.142 (2011). 21575596

[b86] Liu N., Shang J., Tian F., Nishi H. & Abe K. *In vivo* optical imaging for evaluating the efficacy of edaravone after transient cerebral ischemia in mice. Brain Res 1397, 66–75, doi: 10.1016/j.brainres.2011.04.038 (2011). 21571257

[b87] Li L. *et al.* Cerebroside-A provides potent neuroprotection after cerebral ischaemia through reducing glutamate release and Ca(2)(+) influx of NMDA receptors. Int J Neuropsychopharmacol 15, 497–507, doi: 10.1017/S1461145711000654 (2012). 21557879

[b88] Zhang Y. *et al.* Dipyrone inhibits neuronal cell death and diminishes hypoxic/ischemic brain injury. Neurosurgery 69, 942–956, doi: 10.1227/NEU.0b013e318222afb2 (2011). 21552169 PMC4163057

[b89] Jin R. *et al.* Phosphatidylinositol-3-kinase gamma plays a central role in blood-brain barrier dysfunction in acute experimental stroke. Stroke 42, 2033–2044, doi: 10.1161/STROKEAHA.110.601369 (2011). 21546487 PMC3129812

[b90] Custodis F. *et al.* Heart rate contributes to the vascular effects of chronic mental stress: effects on endothelial function and ischemic brain injury in mice. Stroke 42, 1742–1749, doi: 10.1161/STROKEAHA.110.598607 (2011). 21527760

[b91] Oyagi A. *et al.* Forebrain specific heparin-binding epidermal growth factor-like growth factor knockout mice show exacerbated ischemia and reperfusion injury. Neuroscience 185, 116–124, doi: 10.1016/j.neuroscience.2011.04.034 (2011). 21524692

[b92] Jin R. C. *et al.* Glutathione peroxidase-3 deficiency promotes platelet-dependent thrombosis *in vivo*. Circulation 123, 1963–1973, doi: 10.1161/CIRCULATIONAHA.110.000034 (2011). 21518981 PMC3107543

[b93] Berny-Lang M. A. *et al.* Thrombin mutant W215A/E217A treatment improves neurological outcome and reduces cerebral infarct size in a mouse model of ischemic stroke. Stroke 42, 1736–1741, doi: 10.1161/STROKEAHA.110.603811 (2011). 21512172 PMC3115697

[b94] Dolga A. M. *et al.* KCa2 channels activation prevents [Ca^2+^]i deregulation and reduces neuronal death following glutamate toxicity and cerebral ischemia. Cell Death Dis 2, e147, doi: 10.1038/cddis.2011.30 (2011). 21509037 PMC3122061

[b95] Hoffmann U., Lee J. H., Qin T., Eikermann-Haerter K. & Ayata C. Gabapentin reduces infarct volume but does not suppress peri-infarct depolarizations. J Cereb Blood Flow Metab 31, 1578–1582, doi: 10.1038/jcbfm.2011.50 (2011). 21505480 PMC3137475

[b96] Chen J. *et al.* Circulating endothelial progenitor cells and cellular membrane microparticles in db/db diabetic mouse: possible implications in cerebral ischemic damage. Am J Physiol Endocrinol Metab 301, E62–71, doi: 10.1152/ajpendo.00026.2011 (2011). 21505143 PMC3129837

[b97] Teramoto S. *et al.* Exendin-4, a glucagon-like peptide-1 receptor agonist, provides neuroprotection in mice transient focal cerebral ischemia. J Cereb Blood Flow Metab 31, 1696–1705, doi: 10.1038/jcbfm.2011.51 (2011). 21487412 PMC3170947

[b98] Li N. *et al.* Age-related differences in experimental stroke: possible involvement of mitochondrial dysfunction and oxidative damage. Rejuvenation Res 14, 261–273, doi: 10.1089/rej.2010.1115 (2011). 21466386

[b99] Arumugam T. V. *et al.* Evidence that gamma-secretase-mediated Notch signaling induces neuronal cell death via the nuclear factor-kappaB-Bcl-2-interacting mediator of cell death pathway in ischemic stroke. Mol Pharmacol 80, 23–31, doi: 10.1124/mol.111.071076 (2011). 21450930

[b100] Takamiya M. *et al.* Neurological and pathological improvements of cerebral infarction in mice with platinum nanoparticles. J Neurosci Res 89, 1125–1133, doi: 10.1002/jnr.22622 (2011). 21433052

[b101] Shen H. Y. *et al.* Adenosine kinase determines the degree of brain injury after ischemic stroke in mice. J Cereb Blood Flow Metab 31, 1648–1659, doi: 10.1038/jcbfm.2011.30 (2011). 21427729 PMC3137468

[b102] Kim J. H. *et al.* The Traditional Herbal Medicine, Dangkwisoo-San, Prevents Cerebral Ischemic Injury through Nitric Oxide-Dependent Mechanisms. Evid Based Complement Alternat Med 2011, 718302, doi: 10.1155/2011/718302 (2011). 21423636 PMC3057556

[b103] Zhao R., Shi W. Z., Zhang Y. M., Fang S. H. & Wei E. Q. Montelukast, a cysteinyl leukotriene receptor-1 antagonist, attenuates chronic brain injury after focal cerebral ischaemia in mice and rats. J Pharm Pharmacol 63, 550–557, doi: 10.1111/j.2042-7158.2010.01238.x (2011). 21401607

[b104] Liu F. *et al.* Age-related changes in AMP-activated protein kinase after stroke. Age (Dordr) 34, 157–168, doi: 10.1007/s11357-011-9214-8 (2012). 21360073 PMC3260368

[b105] Miyazaki T. *et al.* Distinct effects of tissue-type plasminogen activator and SMTP-7 on cerebrovascular inflammation following thrombolytic reperfusion. Stroke 42, 1097–1104, doi: 10.1161/STROKEAHA.110.598359 (2011). 21350203

[b106] Zhang N. *et al.* Hypoxic preconditioning induced neuroprotection against cerebral ischemic injuries and its cPKCgamma-mediated molecular mechanism. Neurochem Int 58, 684–692, doi: 10.1016/j.neuint.2011.02.007 (2011). 21335048

[b107] Shah Z. A., Nada S. E. & Dore S. Heme oxygenase 1, beneficial role in permanent ischemic stroke and in Gingko biloba (EGb 761) neuroprotection. Neuroscience 180, 248–255, doi: 10.1016/j.neuroscience.2011.02.031 (2011). 21334424 PMC3070771

[b108] Kleinschnitz C. *et al.* Glucocorticoid insensitivity at the hypoxic blood-brain barrier can be reversed by inhibition of the proteasome. Stroke 42, 1081–1089, doi: 10.1161/STROKEAHA.110.592238 (2011). 21330632

[b109] Qian Y. R. *et al.* Neuroprotection by valproic Acid in mouse models of permanent and transient focal cerebral ischemia. Korean J Physiol Pharmacol 14, 435–440, doi: 10.4196/kjpp.2010.14.6.435 (2010). 21311686 PMC3034125

[b110] Liu F. *et al.* Sex differences in the response to poly(ADP-ribose) polymerase-1 deletion and caspase inhibition after stroke. Stroke 42, 1090–1096, doi: 10.1161/STROKEAHA.110.594861 (2011). 21311064 PMC3066270

[b111] Chen H., Kim G. S., Okami N., Narasimhan P. & Chan P. H. NADPH oxidase is involved in post-ischemic brain inflammation. Neurobiol Dis 42, 341–348, doi: 10.1016/j.nbd.2011.01.027 (2011). 21303700 PMC3079796

[b112] Lu Q. *et al.* Betulinic acid protects against cerebral ischemia-reperfusion injury in mice by reducing oxidative and nitrosative stress. Nitric Oxide 24, 132–138, doi: 10.1016/j.niox.2011.01.007 (2011). 21292018

[b113] Bu X. *et al.* Proteomic analysis of cPKCbetaII-interacting proteins involved in HPC-induced neuroprotection against cerebral ischemia of mice. J Neurochem 117, 346–356, doi: 10.1111/j.1471-4159.2011.07209.x (2011). 21291475

[b114] Bahjat F. R. *et al.* Proof of concept: pharmacological preconditioning with a Toll-like receptor agonist protects against cerebrovascular injury in a primate model of stroke. J Cereb Blood Flow Metab 31, 1229–1242, doi: 10.1038/jcbfm.2011.6 (2011). 21285967 PMC3099644

[b115] Wei Y. *et al.* Fingolimod provides long-term protection in rodent models of cerebral ischemia. Ann Neurol 69, 119–129, doi: 10.1002/ana.22186 (2011). 21280082 PMC3200194

[b116] Harada S., Fujita-Hamabe W. & Tokuyama S. Effect of orexin-A on post-ischemic glucose intolerance and neuronal damage. J Pharmacol Sci 115, 155–163 (2011). 21258173 10.1254/jphs.10264FP

[b117] Yilmaz G. *et al.* Selectin-mediated recruitment of bone marrow stromal cells in the postischemic cerebral microvasculature. Stroke 42, 806–811, doi: 10.1161/STROKEAHA.110.597088 (2011). 21257828 PMC3042505

[b118] Zhao Y. & Rempe D. A. Prophylactic neuroprotection against stroke: low-dose, prolonged treatment with deferoxamine or deferasirox establishes prolonged neuroprotection independent of HIF-1 function. J Cereb Blood Flow Metab 31, 1412–1423, doi: 10.1038/jcbfm.2010.230 (2011). 21245873 PMC3130314

[b119] Bradford S. T., Stamatovic S. M., Dondeti R. S., Keep R. F. & Andjelkovic A. V. Nicotine aggravates the brain postischemic inflammatory response. Am J Physiol Heart Circ Physiol 300, H1518–1529, doi: 10.1152/ajpheart.00928.2010 (2011). 21239632 PMC3075028

[b120] Deplanque D., Venna V. R. & Bordet R. Brain ischemia changes the long term response to antidepressant drugs in mice. Behav Brain Res 219, 367–372, doi: 10.1016/j.bbr.2011.01.003 (2011). 21238493

[b121] Ikegame Y. *et al.* Comparison of mesenchymal stem cells from adipose tissue and bone marrow for ischemic stroke therapy. Cytotherapy 13, 675–685, doi: 10.3109/14653249.2010.549122 (2011). 21231804

[b122] Qin L., Kim E., Ratan R., Lee F. S. & Cho S. Genetic variant of BDNF (Val66Met) polymorphism attenuates stroke-induced angiogenic responses by enhancing anti-angiogenic mediator CD36 expression. J Neurosci 31, 775–783, doi: 10.1523/JNEUROSCI.4547-10.2011 (2011). 21228186 PMC3308129

[b123] Valerio A. *et al.* Glycogen synthase kinase-3 inhibition reduces ischemic cerebral damage, restores impaired mitochondrial biogenesis and prevents ROS production. J Neurochem 116, 1148–1159, doi: 10.1111/j.1471-4159.2011.07171.x (2011). 21210815

[b124] Liao Y. *et al.* Neuronal Ca^2+^-activated K^+^ channels limit brain infarction and promote survival. PLoS One 5, e15601, doi: 10.1371/journal.pone.0015601 (2010). 21209897 PMC3012709

[b125] Morrison H. *et al.* The contribution of mannose binding lectin to reperfusion injury after ischemic stroke. Curr Neurovasc Res 8, 52–63 (2011). 21208161 10.2174/156720211794520260 PMC3512100

[b126] Ferrazzano P. *et al.* Inhibiting the Na^+^/H^+^ exchanger reduces reperfusion injury: a small animal MRI study. Front Biosci (Elite Ed.) 3, 81–88 (2011). 21196287 10.2741/e222 PMC3074106

[b127] Moyanova S. G. *et al.* Protective role for type 4 metabotropic glutamate receptors against ischemic brain damage. J Cereb Blood Flow Metab 31, 1107–1118, doi: 10.1038/jcbfm.2010.201 (2011). 21157475 PMC3070971

[b128] Kraft P. *et al.* Deficiency of vasodilator-stimulated phosphoprotein (VASP) increases blood-brain-barrier damage and edema formation after ischemic stroke in mice. PLoS One 5, e15106, doi: 10.1371/journal.pone.0015106 (2010). 21151938 PMC2997079

[b129] Ishiguro M. *et al.* Phosphodiesterase-III inhibitor prevents hemorrhagic transformation induced by focal cerebral ischemia in mice treated with tPA. PLoS One 5, e15178, doi: 10.1371/journal.pone.0015178 (2010). 21151895 PMC2997776

[b130] Li L. & Zuo Z. Glutamate transporter type 3 knockout reduces brain tolerance to focal brain ischemia in mice. J Cereb Blood Flow Metab 31, 1283–1292, doi: 10.1038/jcbfm.2010.222 (2011). 21139629 PMC3099634

[b131] Brait V. H. *et al.* Chemokine-related gene expression in the brain following ischemic stroke: no role for CXCR2 in outcome. Brain Res 1372, 169–179, doi: 10.1016/j.brainres.2010.11.087 (2011). 21138735

[b132] Tureyen K., Bowen K., Liang J., Dempsey R. J. & Vemuganti R. Exacerbated brain damage, edema and inflammation in type-2 diabetic mice subjected to focal ischemia. J Neurochem 116, 499–507, doi: 10.1111/j.1471-4159.2010.07127.x (2011). 21133923 PMC3076322

[b133] Schindowski K. *et al.* Regulation of GDF-15, a distant TGF-beta superfamily member, in a mouse model of cerebral ischemia. Cell Tissue Res 343, 399–409, doi: 10.1007/s00441-010-1090-5 (2011). 21128084 PMC3032194

[b134] Doeppner T. R. *et al.* Acute hepatocyte growth factor treatment induces long-term neuroprotection and stroke recovery via mechanisms involving neural precursor cell proliferation and differentiation. J Cereb Blood Flow Metab 31, 1251–1262, doi: 10.1038/jcbfm.2010.211 (2011). 21119693 PMC3099629

[b135] Bartolini A., Vigliani M. C., Magrassi L., Vercelli A. & Rossi F. G-CSF administration to adult mice stimulates the proliferation of microglia but does not modify the outcome of ischemic injury. Neurobiol Dis 41, 640–649, doi: 10.1016/j.nbd.2010.11.013 (2011). 21111821

[b136] Yoon J. S. *et al.* Pregabalin suppresses calcium-mediated proteolysis and improves stroke outcome. Neurobiol Dis 41, 624–629, doi: 10.1016/j.nbd.2010.11.011 (2011). 21111818 PMC3031782

[b137] Garcia-Yebenes I. *et al.* A mouse model of hemorrhagic transformation by delayed tissue plasminogen activator administration after *in situ* thromboembolic stroke. Stroke 42, 196–203, doi: 10.1161/STROKEAHA.110.600452 (2011). 21106952

[b138] Cai B. *et al.* TAT-mediated delivery of neuroglobin protects against focal cerebral ischemia in mice. Exp Neurol 227, 224–231, doi: 10.1016/j.expneurol.2010.11.009 (2011). 21093435

[b139] Ren X., Akiyoshi K., Vandenbark A. A., Hurn P. D. & Offner H. CD4^+^ FoxP3^+^ regulatory T-cells in cerebral ischemic stroke. Metab Brain Dis 26, 87–90, doi: 10.1007/s11011-010-9226-6 (2011). 21082336 PMC3070853

[b140] Krajewska M. *et al.* Endoplasmic reticulum protein BI-1 modulates unfolded protein response signaling and protects against stroke and traumatic brain injury. Brain Res 1370, 227–237, doi: 10.1016/j.brainres.2010.11.015 (2011). 21075086 PMC3019258

[b141] Inacio A. R., Ruscher K., Leng L., Bucala R. & Deierborg T. Macrophage migration inhibitory factor promotes cell death and aggravates neurologic deficits after experimental stroke. J Cereb Blood Flow Metab 31, 1093–1106, doi: 10.1038/jcbfm.2010.194 (2011). 21063426 PMC3070968

[b142] Fu A., Hui E. K., Lu J. Z., Boado R. J. & Pardridge W. M. Neuroprotection in stroke in the mouse with intravenous erythropoietin-Trojan horse fusion protein. Brain Res 1369, 203–207, doi: 10.1016/j.brainres.2010.10.097 (2011). 21047502

[b143] Morrison H., McKee D. & Ritter L. Systemic neutrophil activation in a mouse model of ischemic stroke and reperfusion. Biol Res Nurs 13, 154–163, doi: 10.1177/1099800410384500 (2011). 21044968 PMC3555226

[b144] Macrez R., Bezin L., Le Mauff B., Ali C. & Vivien D. Functional occurrence of the interaction of tissue plasminogen activator with the NR1 Subunit of N-methyl-D-aspartate receptors during stroke. Stroke 41, 2950–2955, doi: 10.1161/STROKEAHA.110.592360 (2010). 20966414

[b145] Konoeda F. *et al.* Therapeutic effect of IL-12/23 and their signaling pathway blockade on brain ischemia model. Biochem Biophys Res Commun 402, 500–506, doi: 10.1016/j.bbrc.2010.10.058 (2010). 20965150

[b146] Liu N. *et al.* *In vivo* optical imaging of early-stage apoptosis in mouse brain after transient cerebral ischemia. J Neurosci Res 88, 3488–3497, doi: 10.1002/jnr.22489 (2010). 20936709

[b147] Tanaka Y., Tanaka R., Liu M., Hattori N. & Urabe T. Cilostazol attenuates ischemic brain injury and enhances neurogenesis in the subventricular zone of adult mice after transient focal cerebral ischemia. Neuroscience 171, 1367–1376, doi: 10.1016/j.neuroscience.2010.10.008 (2010). 20933581

[b148] Kleinschnitz C. *et al.* Post-stroke inhibition of induced NADPH oxidase type 4 prevents oxidative stress and neurodegeneration. PLoS Biol 8, doi: 10.1371/journal.pbio.1000479 (2010). PMC2943442 20877715

[b149] Chang L. *et al.* Cocaine-and amphetamine-regulated transcript modulates peripheral immunity and protects against brain injury in experimental stroke. Brain Behav Immun 25, 260–269, doi: 10.1016/j.bbi.2010.09.017 (2011). 20869431

[b150] Hurtado O. *et al.* Lack of adrenomedullin, but not complement factor H, results in larger infarct size and more extensive brain damage in a focal ischemia model. Neuroscience 171, 885–892, doi: 10.1016/j.neuroscience.2010.09.021 (2010). 20854881

[b151] Hyakkoku K. *et al.* Toll-like receptor 4 (TLR4), but not TLR3 or TLR9, knock-out mice have neuroprotective effects against focal cerebral ischemia. Neuroscience 171, 258–267, doi: 10.1016/j.neuroscience.2010.08.054 (2010). 20804821

[b152] Liu D. *et al.* Evidence that OGG1 glycosylase protects neurons against oxidative DNA damage and cell death under ischemic conditions. J Cereb Blood Flow Metab 31, 680–692, doi: 10.1038/jcbfm.2010.147 (2011). 20736962 PMC3049522

[b153] Marumo T., Eto K., Wake H., Omura T. & Nabekura J. The inhibitor of 20-HETE synthesis, TS-011, improves cerebral microcirculatory autoregulation impaired by middle cerebral artery occlusion in mice. Br J Pharmacol 161, 1391–1402, doi: 10.1111/j.1476-5381.2010.00973.x (2010). 20735406 PMC3000662

[b154] Menn B. *et al.* Delayed treatment with systemic (S)-roscovitine provides neuroprotection and inhibits *in vivo* CDK5 activity increase in animal stroke models. PLoS One 5, e12117, doi: 10.1371/journal.pone.0012117 (2010). 20711428 PMC2920814

[b155] Oida Y. *et al.* Post-treatment of a BiP inducer prevents cell death after middle cerebral artery occlusion in mice. Neurosci Lett 484, 43–46, doi: 10.1016/j.neulet.2010.08.015 (2010). 20709152

[b156] Xu X. *et al.* Synergistic protective effects of humanin and necrostatin-1 on hypoxia and ischemia/reperfusion injury. Brain Res 1355, 189–194, doi: 10.1016/j.brainres.2010.07.080 (2010). 20682300 PMC3412340

[b157] Qiu J. *et al.* High-mobility group box 1 promotes metalloproteinase-9 upregulation through Toll-like receptor 4 after cerebral ischemia. Stroke 41, 2077–2082, doi: 10.1161/STROKEAHA.110.590463 (2010). 20671243 PMC3066477

[b158] Tai S. H. *et al.* Melatonin inhibits postischemic matrix metalloproteinase-9 (MMP-9) activation via dual modulation of plasminogen/plasmin system and endogenous MMP inhibitor in mice subjected to transient focal cerebral ischemia. J Pineal Res 49, 332–341, doi: 10.1111/j.1600-079X.2010.00797.x (2010). 20663046

[b159] Kraft P., Schwarz T., Meijers J. C., Stoll G. & Kleinschnitz C. Thrombin-activatable fibrinolysis inhibitor (TAFI) deficient mice are susceptible to intracerebral thrombosis and ischemic stroke. PLoS One 5, e11658, doi: 10.1371/journal.pone.0011658 (2010). 20657835 PMC2906507

[b160] Shin J. A. *et al.* Therapeutic effects of resveratrol during acute periods following experimental ischemic stroke. J Neuroimmunol 227, 93–100, doi: 10.1016/j.jneuroim.2010.06.017 (2010). 20655115

[b161] Ehling P. *et al.* Two pore domain potassium channels in cerebral ischemia: a focus on K2P9.1 (TASK3, KCNK9). Exp Transl Stroke Med 2, 14, doi: 10.1186/2040-7378-2-14 (2010). 20646278 PMC2912796

[b162] Ahmad A. S., Ahmad M., Maruyama T., Narumiya S. & Dore S. Prostaglandin D2 DP1 receptor is beneficial in ischemic stroke and in acute exicitotoxicity in young and old mice. Age (Dordr) 32, 271–282, doi: 10.1007/s11357-010-9135-y (2010). 20640551 PMC2926852

[b163] Saleem S., Shah Z. A., Maruyama T., Narumiya S. & Dore S. Neuroprotective properties of prostaglandin I2 IP receptor in focal cerebral ischemia. Neuroscience 170, 317–323, doi: 10.1016/j.neuroscience.2010.06.060 (2010). 20621166 PMC6010185

[b164] Li J., Benashski S. E., Siegel C., Liu F. & McCullough L. D. Adenosine monophosphate activated protein kinase inhibition is protective in both sexes after experimental stroke. Neurosci Lett 482, 62–65, doi: 10.1016/j.neulet.2010.07.007 (2010). 20621158 PMC2937218

[b165] De Meyer S. F. *et al.* Binding of von Willebrand factor to collagen and glycoprotein Ibalpha, but not to glycoprotein IIb/IIIa, contributes to ischemic stroke in mice—brief report. Arterioscler Thromb Vasc Biol 30, 1949–1951, doi: 10.1161/ATVBAHA.110.208918 (2010). 20616311

[b166] Ahmad M. *et al.* The PGE2 EP2 receptor and its selective activation are beneficial against ischemic stroke. Exp Transl Stroke Med 2, 12, doi: 10.1186/2040-7378-2-12 (2010). 20615245 PMC2912268

[b167] Harada S., Fujita-Hamabe W. & Tokuyama S. The importance of regulation of blood glucose levels through activation of peripheral 5′-AMP-activated protein kinase on ischemic neuronal damage. Brain Res 1351, 254–263, doi: 10.1016/j.brainres.2010.06.052 (2010). 20599814

[b168] Atochin D. N. *et al.* Soluble guanylate cyclase alpha1beta1 limits stroke size and attenuates neurological injury. Stroke 41, 1815–1819, doi: 10.1161/STROKEAHA.109.577635 (2010). 20595671 PMC3047459

[b169] Fan Y. Y. *et al.* Activation of the central histaminergic system is involved in hypoxia-induced stroke tolerance in adult mice. J Cereb Blood Flow Metab 31, 305–314, doi: 10.1038/jcbfm.2010.94 (2011). 20588322 PMC3049494

[b170] Kahles T. *et al.* NADPH oxidase Nox1 contributes to ischemic injury in experimental stroke in mice. Neurobiol Dis 40, 185–192, doi: 10.1016/j.nbd.2010.05.023 (2010). 20580928

[b171] Zhang F. *et al.* Enhanced Delivery of Erythropoietin Across the Blood-Brain Barrier for Neuroprotection against Ischemic Neuronal Injury. Transl Stroke Res 1, 113–121, doi: 10.1007/s12975-010-0019-3 (2010). 20577577 PMC2888513

[b172] Harada S. *et al.* Morinda citrifolia fruit juice prevents ischemic neuronal damage through suppression of the development of post-ischemic glucose intolerance. J Nat Med 64, 468–473, doi: 10.1007/s11418-010-0437-2 (2010). 20574728

[b173] Frauenknecht K. *et al.* Neuroprotective effect of Fn14 deficiency is associated with induction of the granulocyte-colony stimulating factor (G-CSF) pathway in experimental stroke and enhanced by a pathogenic human antiphospholipid antibody. J Neuroimmunol 227, 1–9, doi: 10.1016/j.jneuroim.2010.05.043 (2010). 20557950

[b174] Manhas N., Shi Y., Taunton J. & Sun D. p90 activation contributes to cerebral ischemic damage via phosphorylation of Na^+^/H^+^ exchanger isoform 1. J Neurochem 114, 1476–1486, doi: 10.1111/j.1471-4159.2010.06868.x (2010). 20557427 PMC2924815

[b175] Doeppner T. R. *et al.* Transplantation of TAT-Bcl-xL-transduced neural precursor cells: long-term neuroprotection after stroke. Neurobiol Dis 40, 265–276, doi: 10.1016/j.nbd.2010.05.033 (2010). 20554038

[b176] Pfeilschifter W. *et al.* Pyrrolidine dithiocarbamate activates p38 MAPK and protects brain endothelial cells from apoptosis: a mechanism for the protective effect in stroke? Neurochem Res 35, 1391–1401, doi: 10.1007/s11064-010-0197-0 (2010). 20514517

[b177] Migliati E. R. *et al.* Na(+)−K (+)−2Cl (−) cotransport inhibitor attenuates cerebral edema following experimental stroke via the perivascular pool of aquaporin-4. Neurocrit Care 13, 123–131, doi: 10.1007/s12028-010-9376-8 (2010). 20458553

[b178] Yin K. J. *et al.* Peroxisome proliferator-activated receptor delta regulation of miR-15a in ischemia-induced cerebral vascular endothelial injury. J Neurosci 30, 6398–6408, doi: 10.1523/JNEUROSCI.0780-10.2010 (2010). 20445066 PMC2874744

[b179] Shah Z. A. *et al.* The flavanol (−)-epicatechin prevents stroke damage through the Nrf2/HO1 pathway. J Cereb Blood Flow Metab 30, 1951–1961, doi: 10.1038/jcbfm.2010.53 (2010). 20442725 PMC3002885

[b180] Fan Y. *et al.* Endothelial progenitor cell transplantation improves long-term stroke outcome in mice. Ann Neurol 67, 488–497, doi: 10.1002/ana.21919 (2010). 20437584 PMC3026588

[b181] Xu Z. *et al.* Endonuclease G does not play an obligatory role in poly(ADP-ribose) polymerase-dependent cell death after transient focal cerebral ischemia. Am J Physiol Regul Integr Comp Physiol 299, R215–221, doi: 10.1152/ajpregu.00747.2009 (2010). 20427721 PMC2904146

[b182] Lu C. *et al.* Scavenger receptor class-A has a central role in cerebral ischemia-reperfusion injury. J Cereb Blood Flow Metab 30, 1972–1981, doi: 10.1038/jcbfm.2010.59 (2010). 20424635 PMC3002879

[b183] Vest R. S., O’Leary H., Coultrap S. J., Kindy M. S. & Bayer K. U. Effective post-insult neuroprotection by a novel Ca(2+)/calmodulin-dependent protein kinase II (CaMKII) inhibitor. J Biol Chem 285, 20675–20682, doi: 10.1074/jbc.M109.088617 (2010). 20424167 PMC2898334

[b184] Haines B. A., Mehta S. L., Pratt S. M., Warden C. H. & Li P. A. Deletion of mitochondrial uncoupling protein-2 increases ischemic brain damage after transient focal ischemia by altering gene expression patterns and enhancing inflammatory cytokines. J Cereb Blood Flow Metab 30, 1825–1833, doi: 10.1038/jcbfm.2010.52 (2010). 20407461 PMC2948647

[b185] Liu F., Akella P., Benashski S. E., Xu Y. & McCullough L. D. Expression of Na-K-Cl cotransporter and edema formation are age dependent after ischemic stroke. Exp Neurol 224, 356–361, doi: 10.1016/j.expneurol.2010.04.010 (2010). 20406636 PMC2906683

[b186] Kasahara Y. *et al.* Telmisartan suppresses cerebral injury in a murine model of transient focal ischemia. Brain Res 1340, 70–80, doi: 10.1016/j.brainres.2010.03.101 (2010). 20388500

[b187] Jin K., Wang X., Xie L., Mao X. O. & Greenberg D. A. Transgenic ablation of doublecortin-expressing cells suppresses adult neurogenesis and worsens stroke outcome in mice. Proc Natl Acad Sci USA 107, 7993–7998, doi: 10.1073/pnas.1000154107 (2010). 20385829 PMC2867852

[b188] Zhu H. R. *et al.* Icariin protects against brain injury by enhancing SIRT1-dependent PGC-1alpha expression in experimental stroke. Neuropharmacology 59, 70–76, doi: 10.1016/j.neuropharm.2010.03.017 (2010). 20381504

[b189] Sakata Y., Zhuang H., Kwansa H., Koehler R. C. & Dore S. Resveratrol protects against experimental stroke: putative neuroprotective role of heme oxygenase 1. Exp Neurol 224, 325–329, doi: 10.1016/j.expneurol.2010.03.032 (2010). 20381489 PMC2885554

[b190] Abe T. *et al.* Key role of CD36 in Toll-like receptor 2 signaling in cerebral ischemia. Stroke 41, 898–904, doi: 10.1161/STROKEAHA.109.572552 (2010). 20360550 PMC2950279

[b191] Zechariah A., ElAli A. & Hermann D. M. Combination of tissue-plasminogen activator with erythropoietin induces blood-brain barrier permeability, extracellular matrix disaggregation, and DNA fragmentation after focal cerebral ischemia in mice. Stroke 41, 1008–1012, doi: 10.1161/STROKEAHA.109.574418 (2010). 20360548

[b192] Zhang B. *et al.* Estradiol and G1 reduce infarct size and improve immunosuppression after experimental stroke. J Immunol 184, 4087–4094, doi: 10.4049/jimmunol.0902339 (2010). 20304826 PMC3142781

[b193] Kraft P., Schwarz T., Pochet L., Stoll G. & Kleinschnitz C. COU254, a specific 3-carboxamide-coumarin inhibitor of coagulation factor XII, does not protect mice from acute ischemic stroke. Exp Transl Stroke Med 2, 5, doi: 10.1186/2040-7378-2-5 (2010). 20298537 PMC2831840

[b194] Li X. *et al.* Contributions of poly(ADP-ribose) polymerase-1 and -2 to nuclear translocation of apoptosis-inducing factor and injury from focal cerebral ischemia. J Neurochem 113, 1012–1022, doi: 10.1111/j.1471-4159.2010.06667.x (2010). 20236222 PMC2860677

[b195] Kleinschnitz C. *et al.* Early detrimental T-cell effects in experimental cerebral ischemia are neither related to adaptive immunity nor thrombus formation. Blood 115, 3835–3842, doi: 10.1182/blood-2009-10-249078 (2010). 20215643

[b196] Stapels M. *et al.* Polycomb group proteins as epigenetic mediators of neuroprotection in ischemic tolerance. Sci Signal 3, ra15 , doi: 10.1126/scisignal.2000502 (2010). PMC3878609 20197544

[b197] Chen Z. B. *et al.* Human urinary kallidinogenase suppresses cerebral inflammation in experimental stroke and downregulates nuclear factor-kappaB. J Cereb Blood Flow Metab 30, 1356–1365, doi: 10.1038/jcbfm.2010.19 (2010). 20179726 PMC2949229

[b198] Muhammad S., Aller M. I., Maser-Gluth C., Schwaninger M. & Wisden W. Expression of the kcnk3 potassium channel gene lessens the injury from cerebral ischemia, most likely by a general influence on blood pressure. Neuroscience 167, 758–764, doi: 10.1016/j.neuroscience.2010.02.024 (2010). 20167264

[b199] Brait V. H. *et al.* Mechanisms contributing to cerebral infarct size after stroke: gender, reperfusion, T lymphocytes, and Nox2-derived superoxide. J Cereb Blood Flow Metab 30, 1306–1317, doi: 10.1038/jcbfm.2010.14 (2010). 20145655 PMC2949221

[b200] Nagai N. *et al.* Initial brain lesion size affects the extent of subsequent pathophysiological responses. Brain Res 1322, 109–117, doi: 10.1016/j.brainres.2010.01.077 (2010). 20138161

[b201] Jiang S. X. *et al.* Neuropilin 1 directly interacts with Fer kinase to mediate semaphorin 3A-induced death of cortical neurons. J Biol Chem 285, 9908–9918, doi: 10.1074/jbc.M109.080689 (2010). 20133938 PMC2843238

[b202] Ikeda-Matsuo Y. *et al.* Microsomal prostaglandin E synthase-1 and cyclooxygenase-2 are both required for ischaemic excitotoxicity. Br J Pharmacol 159, 1174–1186, doi: 10.1111/j.1476-5381.2009.00595.x (2010). 20128796 PMC2839275

[b203] Mustafa A. K. *et al.* Serine racemase deletion protects against cerebral ischemia and excitotoxicity. J Neurosci 30, 1413–1416, doi: 10.1523/JNEUROSCI.4297-09.2010 (2010). 20107067 PMC2841469

[b204] Heurteaux C. *et al.* Neuroprotective and neuroproliferative activities of NeuroAid (MLC601, MLC901), a Chinese medicine, *in vitro* and *in vivo*. Neuropharmacology 58, 987–1001, doi: 10.1016/j.neuropharm.2010.01.001 (2010). 20064536

[b205] Yin K. J. *et al.* miR-497 regulates neuronal death in mouse brain after transient focal cerebral ischemia. Neurobiol Dis 38, 17–26, doi: 10.1016/j.nbd.2009.12.021 (2010). 20053374 PMC2837803

[b206] Elvers M. *et al.* Impaired alpha(IIb)beta(3) integrin activation and shear-dependent thrombus formation in mice lacking phospholipase D1. Sci Signal 3, ra1 , doi: 10.1126/scisignal.2000551 (2010). PMC3701458 20051593

[b207] Shen Y. *et al.* Carnosine protects against permanent cerebral ischemia in histidine decarboxylase knockout mice by reducing glutamate excitotoxicity. Free Radic Biol Med 48, 727–735, doi: 10.1016/j.freeradbiomed.2009.12.021 (2010). 20043985

[b208] Chi S. *et al.* Baifuzi reduces transient ischemic brain damage through an interaction with the STREX domain of BKCa channels. Cell Death Dis 1, e13, doi: 10.1038/cddis.2009.10 (2010). 21364615 PMC3039290

[b209] Nguemeni C. *et al.* Dietary supplementation of alpha-linolenic acid in an enriched rapeseed oil diet protects from stroke. Pharmacol Res 61, 226–233, doi: 10.1016/j.phrs.2009.12.007 (2010). 20036742

[b210] Vagnerova K. *et al.* Poly (ADP-ribose) polymerase-1 initiated neuronal cell death pathway—do androgens matter? Neuroscience 166, 476–481, doi: 10.1016/j.neuroscience.2009.12.041 (2010). 20035840 PMC3098136

[b211] Keum S. & Marchuk D. A. A locus mapping to mouse chromosome 7 determines infarct volume in a mouse model of ischemic stroke. Circ Cardiovasc Genet 2, 591–598, doi: 10.1161/CIRCGENETICS.109.883231 (2009). 20031639 PMC2889618

[b212] Strecker J. K. *et al.* Effects of G-CSF treatment on neutrophil mobilization and neurological outcome after transient focal ischemia. Exp Neurol 222, 108–113, doi: 10.1016/j.expneurol.2009.12.012 (2010). 20026112

[b213] Samanta J., Alden T., Gobeske K., Kan L. & Kessler J. A. Noggin protects against ischemic brain injury in rodents. Stroke 41, 357–362, doi: 10.1161/STROKEAHA.109.565523 (2010). 20019326 PMC2822348

[b214] Elzer J. G. *et al.* Neuronal estrogen receptor-alpha mediates neuroprotection by 17beta-estradiol. J Cereb Blood Flow Metab 30, 935–942, doi: 10.1038/jcbfm.2009.258 (2010). 20010956 PMC2949189

[b215] Park J. W. *et al.* Green tea polyphenol (−)-epigallocatechin gallate reduces matrix metalloproteinase-9 activity following transient focal cerebral ischemia. J Nutr Biochem 21, 1038–1044, doi: 10.1016/j.jnutbio.2009.08.009 (2010). 19962294

[b216] Kim G. S., Jung J. E., Niizuma K. & Chan P. H. CK2 is a novel negative regulator of NADPH oxidase and a neuroprotectant in mice after cerebral ischemia. J Neurosci 29, 14779–14789, doi: 10.1523/JNEUROSCI.4161-09.2009 (2009). 19940173 PMC2786083

[b217] Felger J. C. *et al.* Brain dendritic cells in ischemic stroke: time course, activation state, and origin. Brain Behav Immun 24, 724–737, doi: 10.1016/j.bbi.2009.11.002 (2010). 19914372 PMC2885548

[b218] Benakis C., Bonny C. & Hirt L. JNK inhibition and inflammation after cerebral ischemia. Brain Behav Immun 24, 800–811, doi: 10.1016/j.bbi.2009.11.001 (2010). 19903520

[b219] Steele A. D. *et al.* Context dependent neuroprotective properties of prion protein (PrP). Prion 3, 240–249 (2009). 19901559 10.4161/pri.3.4.10135 PMC2807698

[b220] Lathia J. D. *et al.* Pivotal role for beta-1 integrin in neurovascular remodelling after ischemic stroke. Exp Neurol 221, 107–114, doi: 10.1016/j.expneurol.2009.10.007 (2010). 19837065

[b221] Leypoldt F. *et al.* Dimethylarginine dimethylaminohydrolase-1 transgenic mice are not protected from ischemic stroke. PLoS One 4, e7337, doi: 10.1371/journal.pone.0007337 (2009). 19809508 PMC2753663

[b222] Liu X., Nakayama S., Amiry-Moghaddam M., Ottersen O. P. & Bhardwaj A. Arginine-vasopressin V1 but not V2 receptor antagonism modulates infarct volume, brain water content, and aquaporin-4 expression following experimental stroke. Neurocrit Care 12, 124–131, doi: 10.1007/s12028-009-9277-x (2010). 19806476

[b223] Mukerji S. S., Rainey R. N., Rhodes J. L. & Hall A. K. Delayed activin A administration attenuates tissue death after transient focal cerebral ischemia and is associated with decreased stress-responsive kinase activation. J Neurochem 111, 1138–1148, doi: 10.1111/j.1471-4159.2009.06406.x (2009). 19780899 PMC2792943

[b224] Karelina K., Norman G. J., Zhang N. & DeVries A. C. Social contact influences histological and behavioral outcomes following cerebral ischemia. Exp Neurol 220, 276–282, doi: 10.1016/j.expneurol.2009.08.022 (2009). 19733169

[b225] Czech B. *et al.* The immunomodulatory sphingosine 1-phosphate analog FTY720 reduces lesion size and improves neurological outcome in a mouse model of cerebral ischemia. Biochem Biophys Res Commun 389, 251–256, doi: 10.1016/j.bbrc.2009.08.142 (2009). 19720050

[b226] Shi Q. *et al.* Adenovirus-mediated brain-derived neurotrophic factor expression regulated by hypoxia response element protects brain from injury of transient middle cerebral artery occlusion in mice. Neurosci Lett 465, 220–225, doi: 10.1016/j.neulet.2009.08.049 (2009). 19703519

[b227] Gui L. *et al.* Adenosine A 2A receptor deficiency reduces striatal glutamate outflow and attenuates brain injury induced by transient focal cerebral ischemia in mice. Brain Res 1297, 185–193, doi: 10.1016/j.brainres.2009.08.050 (2009). 19703429

[b228] Haraguchi T. *et al.* Cerebroprotective action of telmisartan by inhibition of macrophages/microglia expressing HMGB1 via a peroxisome proliferator-activated receptor gamma-dependent mechanism. Neurosci Lett 464, 151–155, doi: 10.1016/j.neulet.2009.08.043 (2009). 19699780

[b229] Zhao B. Q. *et al.* von Willebrand factor-cleaving protease ADAMTS13 reduces ischemic brain injury in experimental stroke. Blood 114, 3329–3334, doi: 10.1182/blood-2009-03-213264 (2009). 19687510 PMC2759655

[b230] Berthet C. *et al.* Neuroprotective role of lactate after cerebral ischemia. J Cereb Blood Flow Metab 29, 1780–1789, doi: 10.1038/jcbfm.2009.97 (2009). 19675565

[b231] Saiki R. *et al.* Intense correlation between brain infarction and protein-conjugated acrolein. Stroke 40, 3356–3361, doi: 10.1161/STROKEAHA.109.553248 (2009). 19661476

[b232] Marsh B. *et al.* Systemic lipopolysaccharide protects the brain from ischemic injury by reprogramming the response of the brain to stroke: a critical role for IRF3. J Neurosci 29, 9839–9849, doi: 10.1523/JNEUROSCI.2496-09.2009 (2009). 19657036 PMC2946887

[b233] Shichita T. *et al.* Pivotal role of cerebral interleukin-17-producing gammadeltaT cells in the delayed phase of ischemic brain injury. Nat Med 15, 946–950, doi: 10.1038/nm.1999 (2009). 19648929

[b234] Wacker B. K., Park T. S. & Gidday J. M. Hypoxic preconditioning-induced cerebral ischemic tolerance: role of microvascular sphingosine kinase 2. Stroke 40, 3342–3348, doi: 10.1161/STROKEAHA.109.560714 (2009). 19644058 PMC2753710

[b235] Koumura A. *et al.* Metallothionein-III knockout mice aggravates the neuronal damage after transient focal cerebral ischemia. Brain Res 1292, 148–154, doi: 10.1016/j.brainres.2009.07.050 (2009). 19635467

[b236] Choe C. U. *et al.* Nitroxyl exacerbates ischemic cerebral injury and oxidative neurotoxicity. J Neurochem 110, 1766–1773, doi: 10.1111/j.1471-4159.2009.06266.x (2009). 19619135

[b237] Jackman K. A., Miller A. A., Drummond G. R. & Sobey C. G. Importance of NOX1 for angiotensin II-induced cerebrovascular superoxide production and cortical infarct volume following ischemic stroke. Brain Res 1286, 215–220, doi: 10.1016/j.brainres.2009.06.056 (2009). 19559686

[b238] Schilling M., Strecker J. K., Ringelstein E. B., Schabitz W. R. & Kiefer R. The role of CC chemokine receptor 2 on microglia activation and blood-borne cell recruitment after transient focal cerebral ischemia in mice. Brain Res 1289, 79–84, doi: 10.1016/j.brainres.2009.06.054 (2009). 19559679

[b239] Shin J. A. *et al.* Ischemic preconditioning-induced neuroprotection is associated with differential expression of IL-1beta and IL-1 receptor antagonist in the ischemic cortex. J Neuroimmunol 217, 14–19, doi: 10.1016/j.jneuroim.2009.06.001 (2009). 19545912 PMC2916648

[b240] Pastor M. D. *et al.* mTOR/S6 kinase pathway contributes to astrocyte survival during ischemia. J Biol Chem 284, 22067–22078, doi: 10.1074/jbc.M109.033100 (2009). 19535330 PMC2755931

[b241] Kim H. W., Cho K. J., Lee B. I., Kim H. J. & Kim G. W. Post-ischemic administration of peptide with apurinic/apyrimidinic endonuclease activity inhibits induction of cell death after focal cerebral ischemia/reperfusion in mice. Neurosci Lett 460, 166–169, doi: 10.1016/j.neulet.2009.05.062 (2009). 19481583

[b242] Zhang W. *et al.* Role of soluble epoxide hydrolase in the sex-specific vascular response to cerebral ischemia. J Cereb Blood Flow Metab 29, 1475–1481, doi: 10.1038/jcbfm.2009.65 (2009). 19471280 PMC2823630

[b243] Zeynalov E., Shah Z. A., Li R. C. & Dore S. Heme oxygenase 1 is associated with ischemic preconditioning-induced protection against brain ischemia. Neurobiol Dis 35, 264–269, doi: 10.1016/j.nbd.2009.05.010 (2009). 19465127 PMC2740718

[b244] Harada S., Fujita W. H., Shichi K. & Tokuyama S. The development of glucose intolerance after focal cerebral ischemia participates in subsequent neuronal damage. Brain Res 1279, 174–181, doi: 10.1016/j.brainres.2009.05.014 (2009). 19445903

[b245] Subramanian S. *et al.* Recombinant T cell receptor ligand treats experimental stroke. Stroke 40, 2539–2545, doi: 10.1161/STROKEAHA.108.543991 (2009). 19443805 PMC2704258

[b246] Zhang Z. *et al.* Baicalin administration is effective in positive regulation of twenty-four ischemia/reperfusion-related proteins identified by a proteomic study. Neurochem Int 54, 488–496, doi: 10.1016/j.neuint.2009.02.005 (2009). 19428793

[b247] Descamps E. *et al.* Experimental stroke protection induced by 4-hydroxybenzyl alcohol is cancelled by bacitracin. Neurosci Res 64, 137–142, doi: 10.1016/j.neures.2009.02.005 (2009). 19428693

[b248] Chen H., Song Y. S. & Chan P. H. Inhibition of NADPH oxidase is neuroprotective after ischemia-reperfusion. J Cereb Blood Flow Metab 29, 1262–1272, doi: 10.1038/jcbfm.2009.47 (2009). 19417757 PMC2733333

[b249] Hua F. *et al.* Differential roles of TLR2 and TLR4 in acute focal cerebral ischemia/reperfusion injury in mice. Brain Res 1262, 100–108, doi: 10.1016/j.brainres.2009.01.018 (2009). 19401158 PMC2722683

[b250] Stowe A. M. *et al.* Neutrophil elastase and neurovascular injury following focal stroke and reperfusion. Neurobiol Dis 35, 82–90, doi: 10.1016/j.nbd.2009.04.006 (2009). 19393318 PMC2708673

[b251] Saleem S., Ahmad A. S., Maruyama T., Narumiya S. & Dore S. PGF(2alpha) FP receptor contributes to brain damage following transient focal brain ischemia. Neurotox Res 15, 62–70, doi: 10.1007/s12640-009-9007-3 (2009). 19384589 PMC6010178

[b252] Zeynalov E. & Dore S. Low doses of carbon monoxide protect against experimental focal brain ischemia. Neurotox Res 15, 133–137, doi: 10.1007/s12640-009-9014-4 (2009). 19384576 PMC2719876

[b253] Doeppner T. R. *et al.* TAT-Hsp70-mediated neuroprotection and increased survival of neuronal precursor cells after focal cerebral ischemia in mice. J Cereb Blood Flow Metab 29, 1187–1196, doi: 10.1038/jcbfm.2009.44 (2009). 19384335

[b254] Nonaka Y. *et al.* Cilostazol protects against hemorrhagic transformation in mice transient focal cerebral ischemia-induced brain damage. Neurosci Lett 452, 156–161, doi: 10.1016/j.neulet.2009.01.039 (2009). 19383431

[b255] Krishnamurthy R. G. *et al.* Asiatic acid, a pentacyclic triterpene from Centella asiatica, is neuroprotective in a mouse model of focal cerebral ischemia. J Neurosci Res 87, 2541–2550, doi: 10.1002/jnr.22071 (2009). 19382233 PMC2941770

[b256] Schilling M., Strecker J. K., Schabitz W. R., Ringelstein E. B. & Kiefer R. Effects of monocyte chemoattractant protein 1 on blood-borne cell recruitment after transient focal cerebral ischemia in mice. Neuroscience 161, 806–812, doi: 10.1016/j.neuroscience.2009.04.025 (2009). 19374937

[b257] Kim H. W., Cho K. J., Park S. C., Kim H. J. & Kim G. W. The adenoviral vector-mediated increase in apurinic/apyrimidinic endonuclease inhibits the induction of neuronal cell death after transient ischemic stroke in mice. Brain Res 1274, 1–10, doi: 10.1016/j.brainres.2009.04.006 (2009). 19374886

[b258] Nonaka Y. *et al.* Combination treatment with normobaric hyperoxia and cilostazol protects mice against focal cerebral ischemia-induced neuronal damage better than each treatment alone. J Pharmacol Exp Ther 330, 13–22, doi: 10.1124/jpet.109.151548 (2009). 19336663

[b259] Zhang M. *et al.* CB2 receptor activation attenuates microcirculatory dysfunction during cerebral ischemic/reperfusion injury. Microvasc Res 78, 86–94, doi: 10.1016/j.mvr.2009.03.005 (2009). 19332079 PMC3319431

[b260] Sevimli S. *et al.* Endogenous brain protection by granulocyte-colony stimulating factor after ischemic stroke. Exp Neurol 217, 328–335, doi: 10.1016/j.expneurol.2009.03.018 (2009). 19332060

[b261] Zhou F. *et al.* Attenuation of neuronal degeneration in thioredoxin-1 overexpressing mice after mild focal ischemia. Brain Res 1272, 62–70, doi: 10.1016/j.brainres.2009.03.023 (2009). 19328186

[b262] Karelina K. *et al.* Social isolation alters neuroinflammatory response to stroke. Proc Natl Acad Sci USA 106, 5895–5900, doi: 10.1073/pnas.0810737106 (2009). 19307557 PMC2667090

[b263] Wang X. *et al.* Methazolamide and melatonin inhibit mitochondrial cytochrome C release and are neuroprotective in experimental models of ischemic injury. Stroke 40, 1877–1885, doi: 10.1161/STROKEAHA.108.540765 (2009). 19299628 PMC2674528

[b264] Wang C. Y. *et al.* Obesity increases vascular senescence and susceptibility to ischemic injury through chronic activation of Akt and mTOR. Sci Signal 2, ra11, doi: 10.1126/scisignal.2000143 (2009). 19293429 PMC2667954

[b265] Liu D., Gharavi R., Pitta M., Gleichmann M. & Mattson M. P. Nicotinamide prevents NAD^+^ depletion and protects neurons against excitotoxicity and cerebral ischemia: NAD^+^ consumption by SIRT1 may endanger energetically compromised neurons. Neuromolecular Med 11, 28–42, doi: 10.1007/s12017-009-8058-1 (2009). 19288225 PMC2677622

[b266] Li R. C. *et al.* Heme-hemopexin complex attenuates neuronal cell death and stroke damage. J Cereb Blood Flow Metab 29, 953–964, doi: 10.1038/jcbfm.2009.19 (2009). 19277051 PMC6015738

[b267] Yuan M. *et al.* Sex differences in the response to activation of the poly (ADP-ribose) polymerase pathway after experimental stroke. Exp Neurol 217, 210–218, doi: 10.1016/j.expneurol.2009.02.012 (2009). 19268668 PMC2672307

[b268] Liu F. *et al.* Sex differences in caspase activation after stroke. Stroke 40, 1842–1848, doi: 10.1161/STROKEAHA.108.538686 (2009). 19265047 PMC2674515

[b269] Saleem S., Shah Z. A., Urade Y. & Dore S. Lipocalin-prostaglandin D synthase is a critical beneficial factor in transient and permanent focal cerebral ischemia. Neuroscience 160, 248–254, doi: 10.1016/j.neuroscience.2009.02.039 (2009). 19254753 PMC2713578

[b270] Harada S. *et al.* Preventive effect of Morinda citrifolia fruit juice on neuronal damage induced by focal ischemia. Biol Pharm Bull 32, 405–409 (2009). 19252286 10.1248/bpb.32.405

[b271] Jin G. *et al.* CD47 gene knockout protects against transient focal cerebral ischemia in mice. Exp Neurol 217, 165–170, doi: 10.1016/j.expneurol.2009.02.004 (2009). 19233173 PMC3722607

[b272] Liu F., Yuan R., Benashski S. E. & McCullough L. D. Changes in experimental stroke outcome across the life span. J Cereb Blood Flow Metab 29, 792–802, doi: 10.1038/jcbfm.2009.5 (2009). 19223913 PMC2748430

[b273] Saleem S., Kim Y. T., Maruyama T., Narumiya S. & Dore S. Reduced acute brain injury in PGE2 EP3 receptor-deficient mice after cerebral ischemia. J Neuroimmunol 208, 87–93, doi: 10.1016/j.jneuroim.2009.01.015 (2009). 19203800 PMC2713583

[b274] Pradillo J. M. *et al.* Toll-like receptor 4 is involved in neuroprotection afforded by ischemic preconditioning. J Neurochem 109, 287–294, doi: 10.1111/j.1471-4159.2009.05972.x (2009). 19200341

[b275] Lambertsen K. L. *et al.* Microglia protect neurons against ischemia by synthesis of tumor necrosis factor. J Neurosci 29, 1319–1330, doi: 10.1523/JNEUROSCI.5505-08.2009 (2009). 19193879 PMC6666095

[b276] Li J. & McCullough L. D. Sex differences in minocycline-induced neuroprotection after experimental stroke. J Cereb Blood Flow Metab 29, 670–674, doi: 10.1038/jcbfm.2009.3 (2009). 19190654 PMC2855881

[b277] Kleinschnitz C. *et al.* Deficiency of von Willebrand factor protects mice from ischemic stroke. Blood 113, 3600–3603, doi: 10.1182/blood-2008-09-180695 (2009). 19182208

[b278] Jackman K. A. *et al.* Reduction of cerebral infarct volume by apocynin requires pretreatment and is absent in Nox2-deficient mice. Br J Pharmacol 156, 680–688, doi: 10.1111/j.1476-5381.2008.00073.x (2009). 19175604 PMC2697707

[b279] Liesz A. *et al.* Regulatory T cells are key cerebroprotective immunomodulators in acute experimental stroke. Nat Med 15, 192–199, doi: 10.1038/nm.1927 (2009). 19169263

[b280] Doeppner T. R. *et al.* TAT-Bcl-x(L) improves survival of neuronal precursor cells in the lesioned striatum after focal cerebral ischemia. Neurobiol Dis 34, 87–94, doi: 10.1016/j.nbd.2008.12.013 (2009). 19167500

[b281] Liu F., Schafer D. P. & McCullough L. D. TTC, fluoro-Jade B and NeuN staining confirm evolving phases of infarction induced by middle cerebral artery occlusion. J Neurosci Methods 179, 1–8, doi: 10.1016/j.jneumeth.2008.12.028 (2009). 19167427 PMC2674851

[b282] Hyakkoku K. *et al.* Thalidomide protects against ischemic neuronal damage induced by focal cerebral ischemia in mice. Neuroscience 159, 760–769, doi: 10.1016/j.neuroscience.2008.12.043 (2009). 19166916

[b283] Kuroki K. *et al.* Effects of transient focal cerebral ischemia in mice deficient in puma. Neurosci Lett 451, 237–240, doi: 10.1016/j.neulet.2009.01.019 (2009). 19159665

[b284] Zhang C., An J., Strickland D. K. & Yepes M. The low-density lipoprotein receptor-related protein 1 mediates tissue-type plasminogen activator-induced microglial activation in the ischemic brain. Am J Pathol 174, 586–594, doi: 10.2353/ajpath.2009.080661 (2009). 19147818 PMC2630566

[b285] Ji S. *et al.* Acute neuroprotection by pioglitazone after mild brain ischemia without effect on long-term outcome. Exp Neurol 216, 321–328, doi: 10.1016/j.expneurol.2008.12.007 (2009). 19146854

[b286] Lee S. R., Kim H. Y., Hong J. S., Baek W. K. & Park J. W. PPARgamma agonist pioglitazone reduces matrix metalloproteinase-9 activity and neuronal damage after focal cerebral ischemia. Biochem Biophys Res Commun 380, 17–21, doi: 10.1016/j.bbrc.2008.12.181 (2009). 19135426

[b287] Lee E. J., Hung Y. C., Chen H. Y., Wu T. S. & Chen T. Y. Delayed treatment with carboxy-PTIO permits a 4-h therapeutic window of opportunity and prevents against ischemia-induced energy depletion following permanent focal cerebral ischemia in mice. Neurochem Res 34, 1157–1166, doi: 10.1007/s11064-008-9892-5 (2009). 19083093

[b288] Hassid B. G. *et al.* Neuronal RAGE expression modulates severity of injury following transient focal cerebral ischemia. J Clin Neurosci 16, 302–306, doi: 10.1016/j.jocn.2007.12.011 (2009). 19071026

[b289] Stetler R. A. *et al.* Hsp27 protects against ischemic brain injury via attenuation of a novel stress-response cascade upstream of mitochondrial cell death signaling. J Neurosci 28, 13038–13055, doi: 10.1523/JNEUROSCI.4407-08.2008 (2008). 19052195 PMC2614130

[b290] Valerio A. *et al.* Leptin is induced in the ischemic cerebral cortex and exerts neuroprotection through NF-kappaB/c-Rel-dependent transcription. Stroke 40, 610–617, doi: 10.1161/STROKEAHA.108.528588 (2009). 19023096

[b291] Muhammad S. *et al.* The HMGB1 receptor RAGE mediates ischemic brain damage. J Neurosci 28, 12023–12031, doi: 10.1523/JNEUROSCI.2435-08.2008 (2008). 19005067 PMC4597312

[b292] Dogrukol-Ak D. *et al.* Isolation of peptide transport system-6 from brain endothelial cells: therapeutic effects with antisense inhibition in Alzheimer and stroke models. J Cereb Blood Flow Metab 29, 411–422, doi: 10.1038/jcbfm.2008.131 (2009). 19002200

[b293] Austinat M. *et al.* Blockade of bradykinin receptor B1 but not bradykinin receptor B2 provides protection from cerebral infarction and brain edema. Stroke 40, 285–293, doi: 10.1161/STROKEAHA.108.526673 (2009). 18988906

[b294] Mito T. *et al.* Decreased damage from transient focal cerebral ischemia by transfusion of zero-link hemoglobin polymers in mouse. Stroke 40, 278–284, doi: 10.1161/STROKEAHA.108.526731 (2009). 18988905 PMC2612731

[b295] Hirt L. *et al.* Protective role of early aquaporin 4 induction against postischemic edema formation. J Cereb Blood Flow Metab 29, 423–433, doi: 10.1038/jcbfm.2008.133 (2009). 18985050

[b296] Jia J. *et al.* Estrogen inhibits Fas-mediated apoptosis in experimental stroke. Exp Neurol 215, 48–52, doi: 10.1016/j.expneurol.2008.09.015 (2009). 18950622 PMC2651740

[b297] Gurer G., Gursoy-Ozdemir Y., Erdemli E., Can A. & Dalkara T. Astrocytes are more resistant to focal cerebral ischemia than neurons and die by a delayed necrosis. Brain Pathol 19, 630–641, doi: 10.1111/j.1750-3639.2008.00226.x (2009). 18947334 PMC8094847

[b298] Meuth S. G. *et al.* The neuroprotective impact of the leak potassium channel TASK1 on stroke development in mice. Neurobiol Dis 33, 1–11, doi: 10.1016/j.nbd.2008.09.006 (2009). 18930826 PMC3714864

[b299] Saleem S., Zhuang H., Biswal S., Christen Y. & Dore S. Ginkgo biloba extract neuroprotective action is dependent on heme oxygenase 1 in ischemic reperfusion brain injury. Stroke 39, 3389–3396, doi: 10.1161/STROKEAHA.108.523480 (2008). 18845796 PMC6010172

[b300] Dong K., Xu W., Yang J., Qiao H. & Wu L. Neuroprotective effects of Tanshinone IIA on permanent focal cerebral ischemia in mice. Phytother Res 23, 608–613, doi: 10.1002/ptr.2615 (2009). 18844253

[b301] Zhou X. Q., Zeng X. N., Kong H. & Sun X. L. Neuroprotective effects of berberine on stroke models *in vitro* and *in vivo*. Neurosci Lett 447, 31–36, doi: 10.1016/j.neulet.2008.09.064 (2008). 18838103

[b302] Koumura A. *et al.* A novel calpain inhibitor, ((1S)-1((((1S)-1-benzyl-3-cyclopropylamino-2,3-di-oxopropyl)amino)carbonyl)-3-met hylbutyl) carbamic acid 5-methoxy-3-oxapentyl ester, protects neuronal cells from cerebral ischemia-induced damage in mice. Neuroscience 157, 309–318, doi: 10.1016/j.neuroscience.2008.09.007 (2008). 18835333

[b303] Mastroiacovo F. *et al.* Induction of the Wnt antagonist, Dickkopf-1, contributes to the development of neuronal death in models of brain focal ischemia. J Cereb Blood Flow Metab 29, 264–276, doi: 10.1038/jcbfm.2008.111 (2009). 18827832

[b304] Sato Y., Meller R., Yang T., Taki W. & Simon R. P. Stereo-selective neuroprotection against stroke with vitamin A derivatives. Brain Res 1241, 188–192, doi: 10.1016/j.brainres.2008.09.020 (2008). 18824156 PMC3829682

[b305] Oyamada N. *et al.* Transplantation of vascular cells derived from human embryonic stem cells contributes to vascular regeneration after stroke in mice. J Transl Med 6, 54, doi: 10.1186/1479-5876-6-54 (2008). 18823569 PMC2567291

[b306] Kilic E. *et al.* ABCC1: a gateway for pharmacological compounds to the ischaemic brain. Brain 131, 2679–2689, doi: 10.1093/brain/awn222 (2008). 18796513

[b307] Morales J. R. *et al.* Activation of liver X receptors promotes neuroprotection and reduces brain inflammation in experimental stroke. Circulation 118, 1450–1459, doi: 10.1161/CIRCULATIONAHA.108.782300 (2008). 18794391

[b308] Belayev L. *et al.* LAU-0901, a novel platelet-activating factor antagonist, is highly neuroprotective in cerebral ischemia. Exp Neurol 214, 253–258, doi: 10.1016/j.expneurol.2008.08.009 (2008). 18793637 PMC2647717

[b309] Yu J., Zhu H., Ko D. & Kindy M. S. Motoneuronotrophic factor analog GM6 reduces infarct volume and behavioral deficits following transient ischemia in the mouse. Brain Res 1238, 143–153, doi: 10.1016/j.brainres.2008.08.053 (2008). 18789909 PMC3275905

[b310] Li W. L. *et al.* Chronic fluoxetine treatment improves ischemia-induced spatial cognitive deficits through increasing hippocampal neurogenesis after stroke. J Neurosci Res 87, 112–122, doi: 10.1002/jnr.21829 (2009). 18711744

[b311] Wong C. H., Bozinovski S., Hertzog P. J., Hickey M. J. & Crack P. J. Absence of glutathione peroxidase-1 exacerbates cerebral ischemia-reperfusion injury by reducing post-ischemic microvascular perfusion. J Neurochem 107, 241–252, doi: 10.1111/j.1471-4159.2008.05605.x (2008). 18691391

[b312] Zeynalov E. *et al.* The perivascular pool of aquaporin-4 mediates the effect of osmotherapy in postischemic cerebral edema. Crit Care Med 36, 2634–2640, doi: 10.1097/CCM.0b013e3181847853 (2008). 18679106 PMC2755627

[b313] Mayanagi K., Katakam P. V., Gaspar T., Domoki F. & Busija D. W. Acute treatment with rosuvastatin protects insulin resistant (C57BL/6J ob/ob) mice against transient cerebral ischemia. J Cereb Blood Flow Metab 28, 1927–1935, doi: 10.1038/jcbfm.2008.81 (2008). 18665182 PMC2632795

[b314] Wang Y. *et al.* Gene inactivation of Na^+^/H^+^ exchanger isoform 1 attenuates apoptosis and mitochondrial damage following transient focal cerebral ischemia. Eur J Neurosci 28, 51–61, doi: 10.1111/j.1460-9568.2008.06304.x (2008). 18662334 PMC2819721

[b315] Nakamura J. *et al.* Targeted disruption of Hsp110/105 gene protects against ischemic stress. Stroke 39, 2853–2859, doi: 10.1161/STROKEAHA.107.506188 (2008). 18658041

[b316] Schwarting S. *et al.* Hematopoietic stem cells reduce postischemic inflammation and ameliorate ischemic brain injury. Stroke 39, 2867–2875, doi: 10.1161/STROKEAHA.108.513978 (2008). 18658037

[b317] Li J., Lu Z., Li W. L., Yu S. P. & Wei L. Cell death and proliferation in NF-kappaB p50 knockout mouse after cerebral ischemia. Brain Res 1230, 281–289, doi: 10.1016/j.brainres.2008.06.130 (2008). 18657523

[b318] Song Y. S. *et al.* The role of Akt signaling in oxidative stress mediates NF-kappaB activation in mild transient focal cerebral ischemia. J Cereb Blood Flow Metab 28, 1917–1926, doi: 10.1038/jcbfm.2008.80 (2008). 18628779 PMC2605287

[b319] Iwanami J. *et al.* Deletion of angiotensin II type 2 receptor attenuates protective effects of bone marrow stromal cell treatment on ischemia-reperfusion brain injury in mice. Stroke 39, 2554–2559, doi: 10.1161/STROKEAHA.107.513275 (2008). 18617657

[b320] Moisse K., Welch I., Hill T., Volkening K. & Strong M. J. Transient middle cerebral artery occlusion induces microglial priming in the lumbar spinal cord: a novel model of neuroinflammation. J Neuroinflammation 5, 29, doi: 10.1186/1742-2094-5-29 (2008). 18606006 PMC2474603

[b321] Shen H., Luo Y., Kuo C. C. & Wang Y. BMP7 reduces synergistic injury induced by methamphetamine and ischemia in mouse brain. Neurosci Lett 442, 15–18, doi: 10.1016/j.neulet.2008.06.052 (2008). 18598737 PMC2493608

[b322] Xu X. *et al.* Neuroprotective effect of humanin on cerebral ischemia/reperfusion injury is mediated by a PI3K/Akt pathway. Brain Res 1227, 12–18, doi: 10.1016/j.brainres.2008.06.018 (2008). 18590709 PMC2575816

[b323] Hua F. *et al.* Preconditioning with a TLR2 specific ligand increases resistance to cerebral ischemia/reperfusion injury. J Neuroimmunol 199, 75–82, doi: 10.1016/j.jneuroim.2008.05.009 (2008). 18585792 PMC2575849

[b324] Nonaka Y., Shimazawa M., Yoshimura S., Iwama T. & Hara H. Combination effects of normobaric hyperoxia and edaravone on focal cerebral ischemia-induced neuronal damage in mice. Neurosci Lett 441, 224–228, doi: 10.1016/j.neulet.2008.06.033 (2008). 18577423

[b325] Denes A., Ferenczi S., Halasz J., Kornyei Z. & Kovacs K. J. Role of CX3CR1 (fractalkine receptor) in brain damage and inflammation induced by focal cerebral ischemia in mouse. J Cereb Blood Flow Metab 28, 1707–1721, doi: 10.1038/jcbfm.2008.64 (2008). 18575457

[b326] Dong W. *et al.* Resveratrol attenuates ischemic brain damage in the delayed phase after stroke and induces messenger RNA and protein express for angiogenic factors. J Vasc Surg 48, 709–714, doi: 10.1016/j.jvs.2008.04.007 (2008). 18572362

[b327] Fujimoto M. *et al.* Tissue inhibitor of metalloproteinases protect blood-brain barrier disruption in focal cerebral ischemia. J Cereb Blood Flow Metab 28, 1674–1685, doi: 10.1038/jcbfm.2008.59 (2008). 18560439

[b328] Varga-Szabo D. *et al.* The calcium sensor STIM1 is an essential mediator of arterial thrombosis and ischemic brain infarction. J Exp Med 205, 1583–1591, doi: 10.1084/jem.20080302 (2008). 18559454 PMC2442636

[b329] Min J. *et al.* Differential neuroprotective effects of carnosine, anserine, and N-acetyl carnosine against permanent focal ischemia. J Neurosci Res 86, 2984–2991, doi: 10.1002/jnr.21744 (2008). 18543335 PMC2805719

[b330] Tang X. N., Cairns B., Cairns N. & Yenari M. A. Apocynin improves outcome in experimental stroke with a narrow dose range. Neuroscience 154, 556–562, doi: 10.1016/j.neuroscience.2008.03.090 (2008). 18511205 PMC2518451

[b331] Kilic U., Kilic E., Matter C. M., Bassetti C. L. & Hermann D. M. TLR-4 deficiency protects against focal cerebral ischemia and axotomy-induced neurodegeneration. Neurobiol Dis 31, 33–40, doi: 10.1016/j.nbd.2008.03.002 (2008). 18486483

[b332] Li J. *et al.* Misoprostol, an anti-ulcer agent and PGE2 receptor agonist, protects against cerebral ischemia. Neurosci Lett 438, 210–215, doi: 10.1016/j.neulet.2008.04.054 (2008). 18472336 PMC2621308

[b333] Haddad M. *et al.* Reduction of hemorrhagic transformation by PJ34, a poly(ADP-ribose)polymerase inhibitor, after permanent focal cerebral ischemia in mice. Eur J Pharmacol 588, 52–57, doi: 10.1016/j.ejphar.2008.04.013 (2008). 18468597

[b334] Mori T. *et al.* Overexpression of human S100B exacerbates brain damage and periinfarct gliosis after permanent focal ischemia. Stroke 39, 2114–2121, doi: 10.1161/STROKEAHA.107.503821 (2008). 18451356 PMC2665284

[b335] Kim E. *et al.* CD36/fatty acid translocase, an inflammatory mediator, is involved in hyperlipidemia-induced exacerbation in ischemic brain injury. J Neurosci 28, 4661–4670, doi: 10.1523/JNEUROSCI.0982-08.2008 (2008). 18448643 PMC2830708

[b336] Li J. M. *et al.* Temporary pretreatment with the angiotensin II type 1 receptor blocker, valsartan, prevents ischemic brain damage through an increase in capillary density. Stroke 39, 2029–2036, doi: 10.1161/STROKEAHA.107.503458 (2008). 18436887

[b337] Oyamada N. *et al.* The role of mineralocorticoid receptor expression in brain remodeling after cerebral ischemia. Endocrinology 149, 3764–3777, doi: 10.1210/en.2007-1770 (2008). 18436714 PMC2488212

[b338] Weise J. *et al.* Overexpression of cellular prion protein alters postischemic Erk1/2 phosphorylation but not Akt phosphorylation and protects against focal cerebral ischemia. Restor Neurol Neurosci 26, 57–64 (2008). 18431006

[b339] Wang X. *et al.* Effects of neuroglobin overexpression on acute brain injury and long-term outcomes after focal cerebral ischemia. Stroke 39, 1869–1874, doi: 10.1161/STROKEAHA.107.506022 (2008). 18403737 PMC2727360

[b340] Polavarapu R., An J., Zhang C. & Yepes M. Regulated intramembrane proteolysis of the low-density lipoprotein receptor-related protein mediates ischemic cell death. Am J Pathol 172, 1355–1362, doi: 10.2353/ajpath.2008.070975 (2008). 18403601 PMC2329844

[b341] Kim H. H. *et al.* Additive effects of statin and dipyridamole on cerebral blood flow and stroke protection. J Cereb Blood Flow Metab 28, 1285–1293, doi: 10.1038/jcbfm.2008.24 (2008). 18382469 PMC2662038

[b342] Zhang W. *et al.* Soluble epoxide hydrolase gene deletion is protective against experimental cerebral ischemia. Stroke 39, 2073–2078, doi: 10.1161/STROKEAHA.107.508325 (2008). 18369166 PMC2654189

[b343] Connolly E. S. Jr., Winfree C. J., Stern D. M., Solomon R. A. & Pinsky D. J. Procedural and strain-related variables significantly affect outcome in a murine model of focal cerebral ischemia. Neurosurgery 38, 523–531, discussion 532 (1996). 8837805 10.1097/00006123-199603000-00021

[b344] Schulte-Herbruggen O., Klehmet J., Quarcoo D., Meisel C. & Meisel A. Mouse strains differ in their susceptibility to poststroke infections. Neuroimmunomodulation 13, 13–18, doi: 10.1159/000092109 (2006). 16612133

[b345] Majid A. *et al.* Differences in vulnerability to permanent focal cerebral ischemia among 3 common mouse strains. Stroke 31, 2707–2714 (2000). 11062298 10.1161/01.str.31.11.2707

[b346] Maeda K., Hata R. & Hossmann K. A. Regional metabolic disturbances and cerebrovascular anatomy after permanent middle cerebral artery occlusion in C57black/6 and SV129 mice. Neurobiol Dis 6, 101–108, doi: 10.1006/nbdi.1998.0235 (1999). 10343325

[b347] Tsuchiya D., Hong S., Kayama T., Panter S. S. & Weinstein P. R. Effect of suture size and carotid clip application upon blood flow and infarct volume after permanent and temporary middle cerebral artery occlusion in mice. Brain Res 970, 131–139 (2003). 12706254 10.1016/s0006-8993(03)02300-x

[b348] Mao Y., Yang G. Y., Zhou L. F., Stern J. D. & Betz A. L. Focal cerebral ischemia in the mouse: description of a model and effects of permanent and temporary occlusion. Brain Res Mol Brain Res 63, 366–370 (1999). 9878831 10.1016/s0169-328x(98)00271-x

[b349] Barber P. A. *et al.* Early T1- and T2-weighted MRI signatures of transient and permanent middle cerebral artery occlusion in a murine stroke model studied at 9.4T. Neurosci Lett 388, 54–59, doi: 10.1016/j.neulet.2005.06.067 (2005). 16055267

[b350] Huang J., Kim L. J., Poisik A., Pinsky D. J. & Connolly E. S. Jr. Does poly-L-lysine coating of the middle cerebral artery occlusion suture improve infarct consistency in a murine model? J Stroke Cerebrovasc Dis 7, 296–301 (1998). 17895104 10.1016/s1052-3057(98)80046-3

[b351] Yuan F. *et al.* Optimizing suture middle cerebral artery occlusion model in C57BL/6 mice circumvents posterior communicating artery dysplasia. J Neurotrauma 29, 1499–1505, doi: 10.1089/neu.2011.2105 (2012). 22054164

[b352] Akamatsu Y., Shimizu H., Saito A., Fujimura M. & Tominaga T. Consistent focal cerebral ischemia without posterior cerebral artery occlusion and its real-time monitoring in an intraluminal suture model in mice. J Neurosurg 116, 657–664, doi: 10.3171/2011.11.JNS111167 (2012). 22196098

[b353] Tureyen K., Vemuganti R., Sailor K. A. & Dempsey R. J. Ideal suture diameter is critical for consistent middle cerebral artery occlusion in mice. Neurosurgery 56, 196–200, discussion 196-200 (2005). 15799811 10.1227/01.neu.0000144490.92966.59

[b354] Macleod M. R. *et al.* Good laboratory practice: preventing introduction of bias at the bench. Stroke 40, e50–52, doi: 10.1161/STROKEAHA.108.525386 (2009). 18703798 PMC2729492

[b355] Schlattmann P. & Dirnagl U. Statistics in experimental cerebrovascular research-comparison of two groups with a continuous outcome variable. J Cereb Blood Flow Metab 30, 474–479, doi: 10.1038/jcbfm.2009.266 (2010). 20051976 PMC2949139

[b356] Sterne J. A. & Davey Smith G. Sifting the evidence-what’s wrong with significance tests? BMJ 322, 226–231 (2001). 11159626 10.1136/bmj.322.7280.226 PMC1119478

[b357] Korevaar D. A., Hooft L. & ter Riet G. Systematic reviews and meta-analyses of preclinical studies: publication bias in laboratory animal experiments. Lab Anim 45, 225–230, doi: 10.1258/la.2011.010121 (2011). 21737463

[b358] Strom J. O., Ingberg E., Theodorsson E. & Theodorsson A. Effects of high and low 17beta-estradiol doses on focal cerebral ischemia: negative results. Sci Rep 3, 3111, doi: 10.1038/srep03111 (2013). 24177749 PMC6505964

[b359] Strom J. O., Strid T. & Hammarstrom S. Disruption of the alox5ap gene ameliorates focal ischemic stroke: possible consequence of impaired leukotriene biosynthesis. BMC Neurosci 13, 146, doi: 10.1186/1471-2202-13-146 (2012). 23194405 PMC3557197

