# Supplementary material for: Method parameters’ impact on mortality and variability in mouse stroke experiments: a meta-analysis
Source: Sci Rep. 2016 Feb 15;6:21086. doi: 10.1038/srep21086 (PMC4753409; doi:10.1038/srep21086)
Supplement: Supplementary Information [file srep21086-s1.pdf]

Method parameters’ impact on mortality and variability in mouse stroke experiments: a meta-analysis

Edvin Ingberg[1]*, Hua Dock[1], Elvar Theodorsson[1], Annette Theodorsson[1,2], Jakob O Ström[1, 3, 4]

1Division of Microbiology and Molecular Medicine, Department of Clinical and Experimental Medicine, Linköping University, Department of Clinical Chemistry, Center for Diagnostics, Region Östergötland

2Division of Neuro and Inflammation Science, Department of Clinical and Experimental Medicine, , Linköping University, Department of Neurosurgery, Anaesthetics, Operations and Specialty Surgery Center, Region Östergötland
3Vårdvetenskapligt Forskningscentrum/Centre for Health Sciences, Örebro University Hospital, County Council of Örebro, Örebro, Sweden

4School of Health and Medical Sciences, Örebro University, Örebro, Sweden.

*Corresponding author. Email: edvin.ingberg@liu.se

Supplementary methods

Category refinement

In the *Other strain* category, the following were included for hypothesis 1A, 2A and 3A: Patchy fur XO, Patchy fur XX, FVB/N, factor V Leiden, Nestin-Cre, SCID and strain not specified. For hypothesis 4A the category also included C3H mice. In the hypotheses 1B, 2B, 3B and 4B analyses the variants ddY, 129S, Mixed C57BL6/129, BALB/c and Strain not specified constituted the *Other strain* category. All strains originating from Swiss mice (e.g. Swiss Webster, ICR, CD-1 and Kunming) were included in the *Swiss* category.

Two separate female categories could be identified in the *Sex* category, one with ovariectomized females and one with females not explicitly ovariectomized. In the *Others* category, groups with mixed sexes and not specified sexes were included. In addition, one group with castrated males were added to the *Others* category for hypotheses 1A, 2A, 3A and 4A while ovariectomized females were added for hypotheses 1B, 2B, 3B and 4B.

The *Age* variable was divided into four categories: *Young* (<2 months), *Adult* (2-12 months), *Elderly* (>12 months) and *Age not specified.*

The number of anesthesia categories were considerably reduced during the refinement process because of similarity between the different variants. All inhalation anesthesia (isoflurane, halothane, sevoflurane, enflurane) formed one group, chloral hydrate alone or in combination formed the second, ketamine alone or in combination the fourth, different members of the benzodiazepine (midazolam, diazepam) and barbiturate (methohexital, thiopenthal, pentobarbital) groups of substances the fifth, tribromoethanol the sixth and finally a category for groups where the anesthesia method was not specified. For hypothesis

4A no control groups represented the *Tribromoethanol* category. Further, for hypothesis 1B, 2B, 3B *Chloral hydrate* and *Tribromoethanol* formed an *Other anesthesia* category and no unspecified anesthesia methods were included. Finally, for hypothesis 4B no control groups represented the *Tribromoethanol* or the *Anesthesia not specified* categories and these were consequently omitted.

The variable *Blood gases/*O_2 *saturation analyzed* was initially extracted as three separate categories (*Blood pH analyzed*, *Blood oxygen saturation analyzed* and *Blood carbon dioxide analyzed*) but these were fused since they were so highly correlated.

Regarding the variable *Type of middle cerebral artery procedure*, all intraluminal filament methods were put together in one category. Note that the effect of different kinds of filaments were analyzed separately in the filament subanalysis. Techniques where the middle cerebral artery was occluded mechanically after craniectomy (clip or electrocauterization) were pooled in a category labelled *Direct, mechanical.* The last two categories were formed by ischemia induced by photothrombosis and different kinds of emboli or clot techniques. For hypothesis 1B, 2B and 3B only two categories were represented, namely occlusion with intraluminal filament or direct craniectomy technique.

Occlusion duration, which theoretically could be seen as a continuous variable was converted into categories to enable comparison with the permanent occlusion option. The category *Short transient* was defined as occlusion less than 60 minutes while more than 60 minutes was considered *Long transient*. Only methods where actions were taken to ensure reperfusion, such as withdrawing the filament, were considered transient. The last category, *Permanent,* included groups were the middle cerebral artery occlusion was induced but not actively reversed.

Twelve types of lesion evaluation were reduced to four main categories. The most frequently used technique, 2,3,5-triphenyltetrazolium chloride staining, defined the first category, while various acidic/basic staining techniques (such as hematoxylin/eosin, thionine and cresyl violet) formed the second. Silver stain were put in a separate category whereas different immunohistochemical methods, neutral red stain and groups without any specified lesion evaluation technique made up the *Other evaluation type* category.

Since a number of studies used more than one exclusion criteria, these were divided into four separate binary variables (*Hemorrhage*, *Deficit score*, *Bad clinical condition* and *Other exclusion criteria*) instead of one variable with four categories. This way one control group could be registered in more than one category. In the *Other exclusion criteria* category, criteria based on hypothermia, oxygen saturation and size of lesion were put. The use of laser Doppler surveillance could also be considered an exclusion criteria, given it is assumed that in studies where this technique was employed, exclusion based on the extent of cerebral perfusion was practiced.

Since the intraluminal filament method is by far the most common procedure for inducing cerebral ischemia (430 out of the total 500 control groups in the current study employed this technique), a separate analysis was performed for these studies (hypotheses 4A and 4B). For this subanalysis, two additional variables were introduced into the model: *Filament coating type* and *Filament insertion vessel*. The variable *Filament coating type* initially comprised ten categories but they were reduced to five after merging of similar coating types. *Silicone* formed a separate category, just like *Poly-L-lysine* did. Different types of glue as well as resin

were put in the *Glue* category while filaments only blunted were considered *Uncoated.* The *Other* category contained filaments coated with Teflon or cases where coating (or lack thereof) was not specified. For hypothesis 4B, only three categories were used since both glue and poly-L-lysine coating were added to the *Other coating* category. This variable was not included in the analyses regarding effect on the mortality outcome measure (hypothesis 1B, 2B, 3B and 4B) since it seems illogical that evaluation type after sacrifice would affect mortality in any way.

Definition of quantitative variables

When data regarding the variable *Weight* was provided as a range (e.g. 20-25 g) the mean value of that range was calculated. For some control groups, weight was not provided at all and to replace these missing values, the mean value of all other control groups was used. *Time after focal ischemia for evaluation of damage* was defined as the time from obstruction of the middle cerebral artery until sacrifice. The first outcome variable, *Infarct size coefficient of variation* was calculated by dividing the standard deviation of the infarct volume by the mean infarct volume. Such definition is strict and applicable irrespective of how the infarct size was presented: as percentage of a hemisphere or as an absolute value in cubic millimeters. The second outcome, *Mortality rate,* was defined as the unintended mortality, from induction of focal cerebral ischemia until sacrifice, as a percentage of the whole control group. This variable was unfortunately only provided for less than 20% of the control groups, why the predictor variables in the analyses regarding this outcome had to be regrouped in some situations to avoid categories represented by less than 5 control groups (see above).

Supplementary tables

Table S1. Regression model for the effect of method parameters' impact on infarct size coefficient of variation (hypotheses 1A, 2A and 3A)

Variable (reference category)	Variable categories	Regression coefficient	0.95 confidence interval for regression coefficient		p-value
Constant	NA	33.2	28.7	37.7	0.000
Strain (C57BL6)	Swiss	-5.7	-11.2	-0.3	0.038
	ddY	14.5	7.4	21.6	0.000
	129	15.9	8.3	23.4	0.000
	Mixed C57BL6/129	22.8	12.5	33.1	0.000
	C3H	9.7	-4.8	24.2	0.189
	BALB/c	-4.0	-17.2	9.1	0.547
	Other strains	5.2	-3.1	13.5	0.221
Sex (Male)	Female	8.7	1.0	16.3	0.027
	Ovx female	-5.3	-14.6	4.0	0.259
	Other sex	3.4	-1.0	7.7	0.132
Type of anesthetic (Inhalation anesthesia)	Chloral hydrate	-1.2	-8.2	5.8	0.737
	Ketamine	15.5	8.7	22.2	0.000
	Benzodiazepines and barbiturates	-4.9	-11.9	2.2	0.177
	Tribromoethanol	4.5	-12.0	21.0	0.591
	Other anesthetic	-5.8	-13.6	2.0	0.142

Awakening during occlusion (No)	[Yes]	4.0	0.1	7.9	0.045
Laser Doppler surveillance (No)	[Yes]	-5.6	-9.7	-1.5	0.007
Blood hemoglobin analyzed (No)	[Yes]	-12.8	-23	-2.6	0.014
Type of middle cerebral artery occlusion procedure (Intraluminal filament)	Direct, mechanical	-0.9	-8.2	6.3	0.797
	Photothrombosis	0.2	-16.0	16.4	0.981
	Emboli/clot	25.9	8.2	43.6	0.004
Occlusion duration (Short transient)	Long transient	-0.9	-4.8	3.0	0.647
	Permanent	-8.6	-15.3	-1.9	0.012
Blinding of infarct size measurement procedure (No)	[Yes]	-5.9	-10.2	-1.6	0.008
Exclusion based on neurological deficit score (No)	[Yes]	6.1	0.5	11.8	0.033
Variables excluded by the statistical software in the backward step of the analysis, due to low explanatory value: Temperature feedback system, Time after ischemia for evaluation of damage, Blood gases/O_2 saturation analyzed, Age, Type of staining, Exclusion based on bad clinical condition, Weight, Blood glucose analyzed, Blood pressure monitored, Exclusion based on hemorrhage, Heart rate monitored, Exclusion based on other criteria.					

**Table S2. Regression model for the effect of method parameters' impact on mortality (hypotheses 1B, 2B and 3B)**					
**Variable (reference category)**	**Variable categories**	**Regression coefficient**	**0.95 confidence interval for regression coefficient**		**p-value**
Constant	NA	29.8	20.6	39.0	0.000
Strain (C57BL6)	Swiss	24.2	16.2	32.2	0.000
	Other strains	-4.2	-10.5	2.1	0.192
Age (Adult)	Young	-10.1	-18.0	-2.2	0.013
	Elderly	15.7	4.1	27.4	0.009
	Age not specified	4.2	-1.5	9.9	0.147
Type of anesthetic	Ketamine	12.4	3.3	21.5	0.009
	Benzodiazepines and barbiturates	-19	26.3	-11.7	0.000

(Inhalation anesthesia)	Other anesthetics	2.5	-9.8	14.7	0.689
Laser Doppler surveillance (No)	[Yes]	-13.4	-20.0	-6.8	0.000
Temperature feedback system (No)	[Yes]	-5.6	-13.9	2.7	0.183
Blood pressure monitored (No)	[Yes]	23.0	14.5	31.6	0.000
Heart rate monitored (No)	[Yes]	-20.3	-32.9	-7.7	0.002
Blood glucose analyzed (No)	[Yes]	-25.6	-35.1	-16.1	0.000
Exclusion based on hemorrhage (No)	[Yes]	-12.2	-22.9	-1.5	0.026
Exclusion based on deficit score (No)	[Yes]	27.3	18.9	35.6	0.000
Variables excluded by the statistical software in the backward step of the analysis, due to low explanatory value: Type of middle cerebral artery occlusion procedure, Occlusion duration, Sex, Exclusion based on other criteria, Exclusion based on bad clinical condition, Time after ischemia for evaluation of damage, Blood gases/O_2 saturation analyzed, Blood hemoglobin analyzed, Weight, Blinding of infarct size measurement procedure, Awakening during occlusion.					

**Table S3. Regression model for the effect of method parameters' impact on infarct size coefficient of variation (hypothesis 4A)**					
**Variable (reference category)**	**Variable categories**	**Regression coefficient**	**0.95 confidence interval for regression coefficient**		**p-value**
Constant	NA	31.3	25.5	37	0.000
Strain	Swiss	-2.0	-8.3	4.2	0.519
	ddY	11.5	4.0	19.1	0.003
	129	15.8	7.8	23.8	0.000
	Mixed C57BL6/129	26.7	13.3	40.1	0.000
	BALB/c	-5.6	-21.4	10.2	0.487
	Other strains	6.8	-2.6	16.1	0.156
Sex (Male)	Female	8.6	0.7	16.6	0.032

	Ovx female	-7.1	-17.3	3.1	0.170
	Other sex	3.8	-1.2	8.8	0.132
Type of anesthetic (inhalation anesthesia)	Chloral hydrate	-0.7	-9.7	8.2	0.875
	Ketamine	11.0	2.8	19.2	0.008
	Benzodiazepines and barbiturates	-13.1	-21.9	-4.3	0.004
	Other anesthetic	-9.3	-17.7	-0.9	0.030
Awakening during occlusion (No)	[Yes]	4.6	0.4	8.7	0.030
Laser Doppler surveillance (No)	[Yes]	-5.2	-9.8	-0.7	0.024
Blood hemoglobin analyzed (No)	[Yes]	-8.4	-22.6	5.8	0.244
Occluding filament type (Silicone)	Poly-L-lysine	-3.8	-11.7	4.2	0.352
	Glue	-3.7	-17.9	10.5	0.608
	Uncoated	4.7	0.8	10.2	0.097
	Other coatings	3.6	-16.2	23.5	0.721
Filament insertion (External carotid artery)	Common carotid artery	1.7	-3.4	6.8	0.514
	Insertion vessel not specified	6.1	1.8	10.3	0.006
Occlusion duration (Short transient)	Long transient	0.6	-3.5	4.8	0.760
	Permanent	-9.8	-17.2	-2.4	0.009
Type of staining (Triphenyl tetrazolium chloride)	Acidic/basic stain	-2.9	-7.9	2.1	0.258
	Silver stain	-13.8	-27.8	0.1	0.051
	Other stains	6.4	-7.2	20.0	0.357
Blinding of infarct size measurement procedure (No)	[Yes]	-8.8	-13.7	-4.0	0.000
Exclusion based on bad clinical condition (No)	[Yes]	15.8	0.3	31.2	0.045
Variables excluded by the statistical software in the backward step of the analysis, due to low explanatory value: Weight, Time after ischemia for evaluation of damage, Blood gases/O_2 saturation analyzed, Temperature feedback system, Age, Blood glucose analyzed,					

Exclusion based on hemorrhage, Exclusion based on other criteria, Heart rate monitored, Blood pressure monitored, Exclusion based on deficit score.

**Table S4. Regression model for the effect of method parameters' impact on mortality (hypothesis 4B)**					
**Variable (reference category)**	**Variable categories**	**Regression coefficient**	**0.95 confidence interval for regression coefficient**		**p-value**
Constant	NA	24.4	14.9	33.8	0.000
Sex (Male)	Female	-10.5	-18.1	-2.9	0.008
	Other sex	-4.9	-12.3	2.4	0.184
Age (Adult)	Young	-14.6	-24	-5.2	0.003
	Elderly	13.4	1.6	25.2	0.027
	Age not specified	5.9	0.1	11.8	0.047
Type of anesthetic (Inhalation anesthesia)	Chloral hydrate	34.7	13.2	56.2	0.002
	Ketamine	19.6	7.2	31.9	0.003
	Benzodiazepines and barbiturates	-6.6	-14.2	0.9	0.085
Laser Doppler surveillance (No)	[Yes]	-1.9	-9.7	5.9	0.629
Temperature feedback system (No)	[Yes]	-8.5	-22.4	5.4	0.224
Heart rate monitored (No)	[Yes]	-16.6	-31.3	-1.9	0.028
Blood gases/O_2 saturation analyzed (No)	[Yes]	11.4	1.8	21.0	0.021
Blood hemoglobin analyzed (No)	[Yes]	-38.0	-56.6	-19.6	0.000
Blood glucose monitored (No)	[Yes]	-8.5	-22.4	5.4	0.224
Filament insertion (External carotid artery)	Common carotid artery	-11.8	-19.5	-4.2	0.003
	Insertion vessel not specified	-3.4	-10.4	3.5	0.323
Blinding of infarct size measurement	[Yes]	16.0	9.3	22.7	0.000

procedure (No)					
Exclusion based on hemorrhage (No)	[Yes]	-21.4	-32.0	-10.8	0.000
Exclusion based on neurological deficit score (No)	[Yes]	35.9	25.0	46.7	0.000
Exclusion based on bad clinical condition (No)	[Yes]	-16.8	-34.5	0.8	0.061
Variables excluded by the statistical software in the backward step of the analysis, due to low explanatory value: Occluding filament type, Exclusion based on other criteria, Occlusion duration, Time after ischemia for evaluation of damage, Blood pressure monitored, Weight, Awakening during occlusion.					
